# Autonomous discovery of emergent morphologies in directed self-assembly of block copolymer blends

**DOI:** 10.1126/sciadv.add3687

**Published:** 2023-01-13

**Authors:** Gregory S. Doerk, Aaron Stein, Suwon Bae, Marcus M. Noack, Masafumi Fukuto, Kevin G. Yager

**Affiliations:** ^1^ Center for Functional Nanomaterials, Brookhaven National Laboratory, Upton, NY 11973, USA.; ^2^ The Center for Advanced Mathematics for Energy Research Applications (CAMERA), Lawrence Berkeley National Laboratory, Berkeley, CA 94720, USA.; ^3^ National Synchrotron Light Source II, Brookhaven National Laboratory, Upton, NY 11973, USA.

## Abstract

The directed self-assembly (DSA) of block copolymers (BCPs) is a powerful approach to fabricate complex nanostructure arrays, but finding morphologies that emerge with changes in polymer architecture, composition, or assembly constraints remains daunting because of the increased dimensionality of the DSA design space. Here, we demonstrate machine-guided discovery of emergent morphologies from a cylinder/lamellae BCP blend directed by a chemical grating template, conducted without direct human intervention on a synchrotron x-ray scattering beamline. This approach maps the morphology-template phase space in a fraction of the time required by manual characterization and highlights regions deserving more detailed investigation. These studies reveal localized, template-directed partitioning of coexisting lamella- and cylinder-like subdomains at the template period length scale, manifesting as previously unknown morphologies such as aligned alternating subdomains, bilayers, or a “ladder” morphology. This work underscores the pivotal role that autonomous characterization can play in advancing the paradigm of DSA.

## Teaser

Combinatorial sampling, synchrotron X-ray scattering, and autonomous steering enable rapid discovery of self-assembled phases.

## INTRODUCTION

An overarching goal of research in self-assembly is to create arbitrarily elaborate and customizable objects with prescribed hierarchical control down to the nanoscale or even molecular scale. Encoding the requisite complex organizational information, however, entails the use of concomitantly more complex building blocks or constraints, sacrificing in part, the appeal of self-assembly as a synthesis strategy (*1*). This trade-off is exemplified in block copolymers (BCPs), where the macromolecular architecture reliably encodes the emergent self-assembled morphology, correlating morphological customizability with synthetic complexity (*2*). This interdependence can be overcome in part by blending BCPs with other polymers or nanomaterials that selectively segregate to one block (*3*–*11*) or layering BCPs with different domain sizes or morphologies (*12*–*17*). Meanwhile, epitaxial registration of assembled BCP domains to chemical or topographical templates in directed self-assembly (DSA) can impose local control over BCP chain distortion, domain orientation, or pattern transferability to generate non-native pattern symmetries in two and three dimensions (*18*–*22*). These approaches avoid the synthesis of complex polymers but introduce their own complexities with respect to process control, template design, and composition selection. Accelerating progress in their use hinges upon exploring the relevant high-dimensional parameter spaces more efficiently and discovering the unifying principles that dictate how and why particular assembly motifs emerge.

Previously, we have demonstrated an approach to selectively enhance and control the complexity of nanopatterns formed through DSA by using a mixture of BCPs that acts as a responsive blend (*23*). Specifically, we used a 1:1 blend of cylindrical and lamellar diblock copolymers of near-equal molar mass, which self-assembles to form coexisting line and dot patterns in thin films (*24*). Using a chemical grating template to order and align the BCP blend pattern through DSA, competing influences of polymer chain distortion and preferential wetting can be exploited to locally select between line and dot patterns simply by adjusting the grating template pitch and/or linewidth. This locally selective DSA can even be extended to single-domain resolution with precise registry between the template and the self-assembled pattern to form a single line in an ordered hexagonal array of dots, for instance (*23*). Because of the challenges of manual characterization and analysis, however, the prior investigation was restricted to a narrow region of the template parameter space around where the template pitch (Λ) matches the natural domain spacing of the BCP blend (*L*_0_). In this narrow template range, the resulting line and dot patterns are effectively two-dimensional; more complex assembly behavior, including the emergence of three-dimensional morphologies, may be possible as the template parameter space is widened. Here, we investigate the assembly of this same blend on chemical patterns spanning a much larger range of line widths and pitches, including pitches more than two times the equilibrium self-assembled spacing (Λ ≥ 2*L*_0_). To bypass the laborious, time-consuming, and often-confusing enterprise of characterization and analysis by exhaustive electron microscopy, we instead probe the system autonomously using synchrotron x-ray scattering. Without direct human intervention, we systematically map the self-assembly behavior in multiple quantitative dimensions across the entire chemical pattern space.

The results of this autonomous mapping reveal an intriguing capacity for the blend to form unusual three-dimensional morphologies, whose unit cells exhibit local partitioning into nominally cylinder-like and lamella-like subdomains, all in registry with the underlying chemical template. These emergent three-dimensional morphologies depend sensitively on the chemical pattern dimensions. At incommensurate values of Λ between *L*_0_ and 2*L*_0_, this can yield subdomain bilayers exhibiting preferential top subdomain alignment along specific skew angles with respect to the grating direction. Meanwhile, in the vicinity of Λ ≈ 2*L*_0_, subdomain partitioning manifests as either line patterns composed of alternating subdomains aligned along the grating direction or a “ladder” morphology consisting of lamella-like “rails” and cylinder-like “rungs.” To the best of our knowledge, neither the ladder, skew, nor alternating morphologies have been observed in previous studies. These findings underscore the immense potential for pairing combinatorial sampling with autonomous characterization to empower researchers and to accelerate the discovery of approaches for generating designer hierarchical self-assembled morphologies.

## RESULTS

**Fig. 1.**
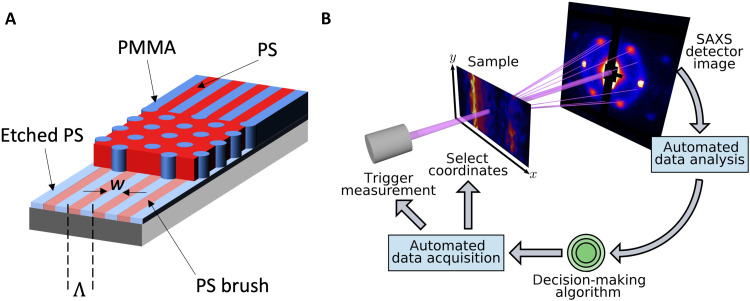
Schematics of the experimental approach. (**A**) DSA of a 1:1 blend of cylindrical and lamellar PS-*b*-PMMA BCPs is achieved using a grating chemical surface pattern with hydrophilic stripes of width *w* on a pitch Λ, etched into a surface-grafted PS brush. (**B**) Autonomous SAXS measurement schematic. A motion system allows the instrument to select any (*x*, *y*) coordinate on the surface of the combinatorial sample for measurement. The SAXS pattern at each position is fed into a data analysis pipeline. A decision-making algorithm based on Gaussian process (GP) methods then selects the next coordinate for measurement.

Our strategy to accelerate the discovery of self-assembled phases comprises three synergistically linked elements. The first is the fabrication of a combinatorial array sample with systematically varied chemical template dimensions, by which one can survey a large parameter space without resorting to serial sample creation. Following our previous work (*23*), the assembly process starts with a chemical pattern fabricated using polymer brush grafting, electron beam lithography, and oxygen plasma etching. The chemical pattern is a simple one-dimensional grating consisting of alternating hydrophobic polystyrene (PS) brush stripes and hydrophilic (ostensibly silicon oxide) stripes of width *w* with a template pitch Λ (Fig. 1A). The sample comprises a contiguous array of grating chemical patterns, each pattern being a 60 μm–by–60 μm square field with lines oriented parallel to the sample *y* axis. Two defining features of the gratings are systematically varied across this combinatorial array. The electron beam patterning dose increases with *y_c_*, effectively increasing *w*, while Λ varies from 30 to 130 nm with *x_c_*, where the “*c*” subscript indicates positions in the patterned combinatorial array. This combinatorial array, depicted schematically in fig. S1, increases the design space with respect to our previous work by a factor of five in terms of Λ alone. As in our previous report, cylinder- and lamellae-forming PS-*block*-poly(methyl methacrylate) (PS-*b*-PMMA) with nearly equivalent molar mass were blended in equal parts by weight in toluene solution. Films about 40 nm thick were spin-casted from solution onto the chemical pattern. The film was annealed overnight under vacuum to enable self-assembly of the BCP blend thin film with an isotropic (undirected) natural period (*L*_0_) of ~54 nm. To enhance contrast for x-ray scattering and to enable electron microscopy of the domain structure, PMMA was selectively infiltrated with Al_2_O_3_ and the polymer was subsequently removed using oxygen plasma treatment, generating inorganic replicas of the assembled PMMA domains. Removal of the polymer would cause collapse for morphologies consisting of isolated domains (e.g., spheres); the prevalent vertical orientation and interconnectivity of the inorganic replicas prevent this in morphological structures observed here. Details of the template fabrication, assembly, and infiltration processes can be found in Materials and Methods.

The second element of our approach is the use of microfocused transmission small-angle x-ray scattering (SAXS) at a synchrotron x-ray scattering beamline, in which measurements performed with an area of tens of micrometers provide a wealth of structural information on the time scale of seconds. This provides the high-resolution characterization needed to understand complex assembly behavior at speeds required to explore the design space efficiently. SAXS measurements were performed in transmission geometry, as shown schematically in Fig. 1B, at the Soft Matter Interfaces (SMI) beamline at the National Synchrotron Light Source II (NSLS-II). To avoid unwanted averaging across adjacent patterning fields, the x-ray beam was focused to a ~2-μm vertical by ~25-μm horizontal spot on the sample. The 200-μm-thick Si wafer substrates and the high x-ray contrast provided by the alumina replicas of PMMA domain structures ensured sufficient signal that required only 2 s per measurements. Compared with a more conventional approach to characterization, e.g., systematic scanning electron microscopy by a capable operator, synchrotron SAXS offers major improvements in measurement throughput. Assuming 90 s per image, using SAXS at a synchrotron can provide a ~45× boost in characterization speed.

The third element of our strategy is the use of autonomous characterization methods, wherein a machine-learning algorithm selects measurements based on a continually updating analysis of the existing dataset. As described in detail in the Materials and Methods and in previous publications (*25*–*28*), the autonomous x-ray measurements and analysis were performed using a fully automated, closed-loop workflow that combines sample positioning, data collection, real-time data analysis, and machine-guided selection of subsequent measurement locations within the pattern array. The control algorithm was a Gaussian process (GP) regression method (*29*), which constructs an interpolated surrogate model for the experimental parameter space, along with a corresponding uncertainty model. Over the course of the experiment, we switched between maximizing three different objective functions [*F*(*x_c_*, *y_c_*)]. The first is defined as the GP posterior variance of the scattering intensity (of the peak associated with the BCP), $F(x_{c},y_{c})=\sigma_{GP}^{2}(x_{c},y_{c})$, which leads to efficient exploration of the input space without any particular focus. This objective function places new measurement points wherever model uncertainty is highest, thus maximizing knowledge gain per measurement. The second objective function, defined as *F*(*x_c_*, *y_c_*) = 3σ_GP_(*x_c_*, *y_c_*) + *m*_GP_(*x_c_*, *y_c_*)σ_GP_(*x_c_*, *y_c_*), where *m*_GP_ is the GP posterior mean of the scattering intensity, balances maximum signal and uncertainty while simultaneously preventing the data acquisition from getting stuck in specific regions of the parameter space. This mode provides a balance between focusing on regions of high scattering intensity while also continuing to explore the parameter space. The third objective function drew the focus onto regions with high uncertainty among scattering peak heights in the 30°, 60°, and 90° azimuthal directions. This objective function is given by

$$F(x_{c},y_{c})=\mathrm{mean}\{{|a(x_{c},y_{c})-\mathrm{mean}[a(x_{c},y_{c})]|}^{2}\}$$ (1)

where **a**(*x_c_*, *y_c_*) is a vector of the positive peak height posterior means in the three directions. This objective function focuses attention on areas deemed “interesting” by the experimenter in the sense that they are regions with notable differences in the scattering of the different morphological populations. The ability to shift between different acquisition strategies, while always leveraging all previously collected data in modeling and thus prediction, is a useful feature of the human-supervised approach described here and enabled efficient data collection. Overall, the GP method implements Bayesian modeling and thus allows data collection that accounts for uncertainty. By selecting locations with large model uncertainty for subsequent measurements, the algorithm maximizes information gain per measurement. Including a term in objective functions that scales with signal intensity emphasizes data collection in regions with good structural order. Last, inclusion of multiple signals (for different azimuthal angles) allows the model to consider the ordering of multiple populations simultaneously and thus to minimize the uncertainty in the distributions of these distinct populations.

By avoiding oversampling of areas with low uncertainty, as would occur in a grid search, the GP algorithm rapidly highlights areas meriting further scientific investigation. This benefit becomes critically important as parameter spaces increase in size and dimensionality or as individual measurements become more costly (e.g., require longer acquisition times) (*27*). In the work presented here, synthetic tests comparing GP regression to a simulated grid search at the same number of measurements reveal that the autonomous algorithm achieves both a faster reduction in error and a lower overall error in modeling the experimental parameter space. The surrogate model shows that the autonomous GP algorithm provides a complete model of the parameter space in a fraction of the time (see fig. S2 in the Supplementary Materials). Moreover, while synchrotron x-ray scattering affords notable advantages in certain contexts (x-ray scattering may be necessary to resolve nanoscale three-dimensional morphologies), the GP regression algorithm is agnostic to the characterization approach. It could be applied equally well, for example, to electron microscope images acquired through critical dimension scanning electron microscopy, a rapid and automated characterization method in common use within the nanoelectronics industry. Fourier transforms of the resultant scanning electron micrographs (SEMs) could be analyzed by automated fitting procedures similar to those used here.

**Fig. 2.**
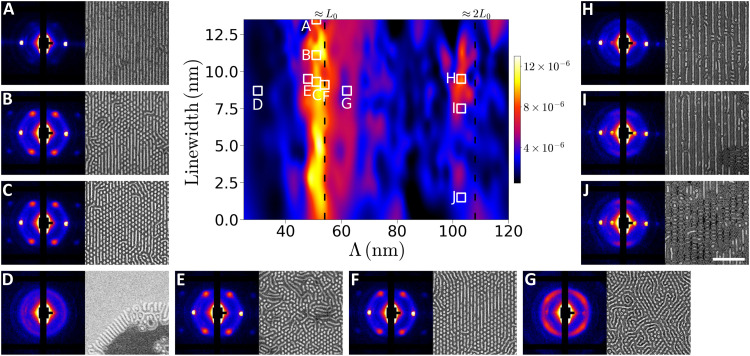
Emergent morphologies within the DSA design space. At the center is a map of scattering prefactor intensity (*p*) as a function of Λ and *w*. Exemplar SAXS patterns, accompanied by corresponding SEMs, indicate regions of the design space dominated by self-assembled line (**A**), hexagonal or line (**B**, **C**, **E**, and **F**), isotropic (**D**), skew (**G**), and alternating cylinders and lamellae or ladder (**H** to **J**) patterns. These pattern types are described in more detail in the main text. The white scale bar denoting 500 nm in (J) applies to all SEMs.

Our automated analysis pipeline provides several signals that can be used as the quantity of interest for autonomous control, including scattering peak height, position, or width (which measure the amount of ordered material, repeat-spacing, and the spatial correlation length, respectively). As noted above, the scattering peak height (i.e., the prefactor from a Gaussian fit to the first-order peak arising from the BCP ordering) along different azimuthal directions was selected as the input signal for the work shown here. The GP model for the fitted prefactor (*p*) of the primary scattering peak is plotted as a function of Λ and *w* in Fig. 2. We note that the values of *w* shown here are simulated as described in Materials and Methods, and absolute values may be different after oxygen plasma etching, although the trends are preserved. The GP model extrapolates slightly to the lowest and highest *w* values, as shown in fig. S3. Similar maps of domain repeat spacing (*d*_0_) and spatial correlation length (ξ) can be generated on the basis of the fit of the primary scattering peak. These maps represent specific populations of pattern types based on the choice of an azimuthal sector average (see fig. S4).

As a representation of the degree of order and alignment along the grating direction (perpendicular to Λ), the spatial distribution of *p* immediately captures salient features of the samples. Two high-intensity vertical bands signaling optimal alignment and ordering are present at Λ just larger than ~50 and ~100 nm. An informed researcher might anticipate these bands at Λ ≈ *L*_0_ and Λ ≈ 2*L*_0_, as BCPs are expected to align and register with the template at these pitch dimensions to minimize both the interfacial and elastic free energies in the BCP thin film (*23*, *30*, *31*). Counter to expectations, however, *p* is highest at Λ approximately ~4% less than *L*_0_ or 2*L*_0_, which can be attributed to the inherent responsivity of the blend (compared to a neat BCP) and the lower free-energy penalty for chain compression versus tension (*32*). The autonomous workflow found this feature independently of human experimenters. In addition, the autonomous workflow revealed trends that are not expected on the basis of heuristic guidance, such as asymmetric intensity toward larger pitches in the vicinity of Λ ≥ *L*_0_ and a highly dose-dependent intensity in the range of Λ ≈ 2*L*_0_. These features motivated detailed and targeted additional investigation, as discussed below.

BCP domain alignment to the chemical pattern is maximized when the Λ is nearly commensurate with the *L*_0_. Consistent with our previous report (*23*), holding Λ near ~*L*_0_ while decreasing the dose results in a transition from assembled line patterns to hexagonal dot patterns (Fig. 2, A to C). At all *w* values for Λ < 44 nm, the morphological pattern is predominantly isotropic, as evidenced by the azimuthally uniform SAXS pattern obtained from the exemplar measurement in Fig. 2D. The corresponding SEMs obtained from the same approximate location show that the morphology is composed of terraced lamellae, with lines orthogonal to the terrace edges, which likely include a combination of both cylindrical and lamellar chains. At these chemical pattern pitches, the BCP blend cannot align in registry with the pattern without excessive chain deformation; instead, the patterned brush can be considered a “compositionally random” brush (*33*) that presents a net PS-preferential interface for the blend given that the calculated duty cycles (*w*/Λ) patterned by our electron beam lithography process are less than 30% across the entire array. Hexagonal dot patterns are apparent at Λ just slightly less than *L*_0_ (Fig. 2E), but the conflicting influences of template wetting and chain compression frustrate ordered alignment, leading to an enhanced level of defectivity. As Λ is increased to values above ~*L*_0_, however, the hexagonal dot patterns (Fig. 2F) give way to a different pattern type in which PMMA domains form partial line patterns that skew within the substrate plane at specific angles with respect to the grating axis, as exemplified in Fig. 2G.

A strong dependence of the magnitude of *p* with respect to dose is observed near Λ ≈ 2*L*_0_, a feature not observed at Λ ≈ *L*_0_. The reason for this can be ascertained by inspection of SAXS patterns and SEMs acquired at Λ ≈ 2*L*_0_, exemplars of which are shown for various doses in Fig. 2 (H to J). Higher doses (Fig. 2, H and I) are characterized by line patterns parallel with the grating direction. The lines exhibit alternating high and low contrast in the corresponding SEMs. As detailed later, we hypothesize that these lines are alternating subdomains enriched with cylindrical or lamellar chains. On the other hand, a completely different morphology that resembles a ladder with rails along the grating direction and rungs orthogonal to it emerges at lower doses (Fig. 2J). This ladder morphology can be considered as a more complex partitioning of cylinder-like and lamella-like subdomains, much like what is observed with the alternating lamella-like and cylinder-like subdomains at high doses, but in which the cylinder-like subdomains orient perpendicularly to the grating direction to satisfy commensurability constraints. A larger set of exemplary SAXS patterns and SEMs are presented in figs. S5 and S6.

**Fig. 3.**
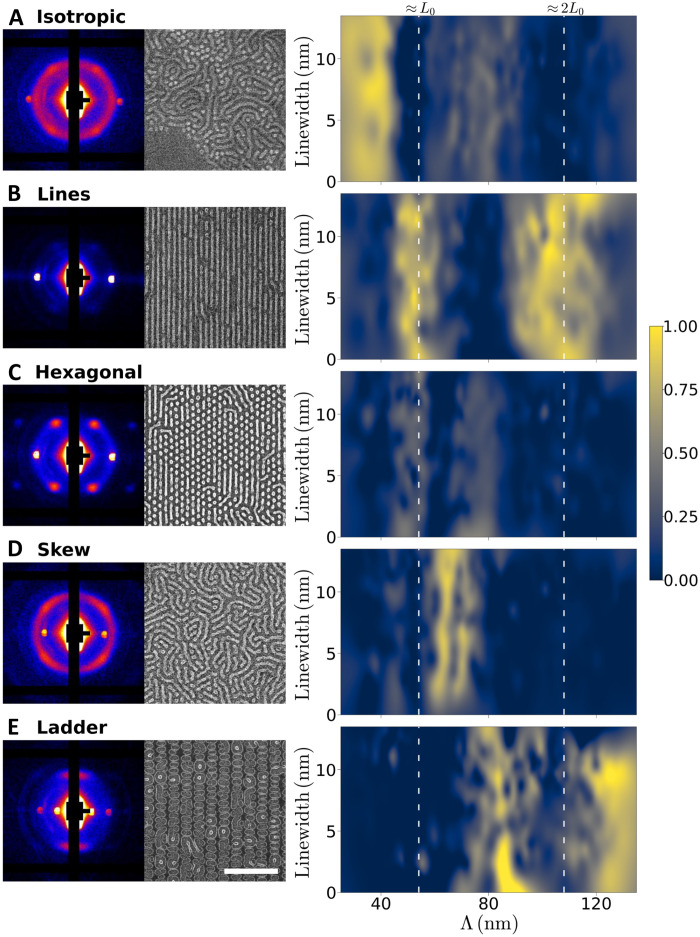
Weighted contributions from specific pattern motifs within the template design space based on fitting the azimuthal scattering data. Exemplar SAXS patterns and SEMs (left) corresponding to intensity maps (right) are shown for self-assembled isotropic (**A**), line (**B**), hexagonal (**C**), skew (**D**), and ladder (**E**) patterns. The white scale bar denoting 500 nm in (E) applies to all SEMs.

On the basis of the observations above, five distinct types of patterns are observed within the template area: isotropic, lines, hexagonal (dots), skew, and ladder. The scattering intensity along the azimuthal direction can be decomposed into a weighted set of contributions corresponding to the identified morphology types. From this, we can construct maps of each patterning motif within the Λ versus *w* design space, as shown in Fig. 3 (details about how these interpolated maps are prepared may be found in Materials and Methods). These intensity maps of selected SAXS signals reveal notable trends in the way the blend self-assembles in response to the underlying template. As expected, isotropic patterns (Fig. 3A) dominate for Λ < *L*_0_, where the substrate surface is compositionally random, while line patterns (Fig. 3B) are most prominent when the template pitch is nearly commensurate with the domain spacing (Λ ≈ *L*_0_ or 2*L*_0_). Hexagonal patterns (Fig. 3C) overlap with the line patterns around Λ ≈ *L*_0_. While this analysis does not show the dose-dependent transition between hexagonal and line patterns, it does show that hexagonal patterns are also prominent in region of Λ≥ *L*_0_. Skew patterns (Fig. 3D) emerge as Λ is increased to just larger than *L*_0_ and recede in prominence as Λ is further increased. The ladder pattern (Fig. 3E) replaces the skew pattern with increasing pitch and becomes the dominant pattern in the array for larger pitches except in proximity to Λ ≈ 2*L*_0_. These trends demonstrate that the blends are highly responsive to the template chemical pattern in unusual ways that would be difficult to predict, find, and understand by a conventional, manual experiment paradigm.

**Fig. 4.**
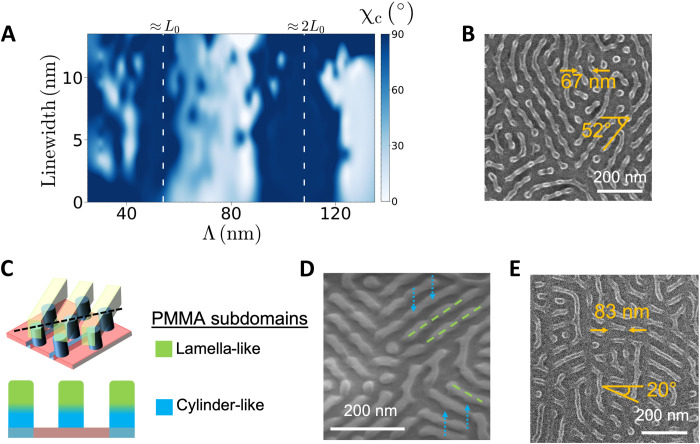
Bilayer and complex morphologies in the skew region of the template parameter space. (**A**) Map of χ*_c_* across the template design space. Patterns are aligned with the grating direction at χ*_c_* = 90° and against the grating direction at χ*_c_* = 0°. A continuous variation in χ*_c_* is apparent in the region identified with the skew morphology within the range of *L*_0_ ≤ Λ ≤ 2*L*_0_. (**B**) SEM of a skew pattern showing vertical hexagonal posts connected by lines at angles similar to those of χ*_c_* measured by SAXS patterns for the first population. (**C**) Schematic of the proposed bilayer morphology responsible for skew patterns. Lamella-like subdomains in the top layer (green) are transparent in the top view. The cross section is taken through the vertical plane along the dashed black line. Green and blue represent lamella-like (i.e., enriched with symmetric BCP chains) and cylinder-like (i.e., enriched with PMMA-minority BCP chains) PMMA subdomains, respectively. (**D**) SEM of a skew pattern taken at a 45° tilt angle. Blue dotted arrows and green dashed lines indicate the inferred positions of cylinder-like and lamella-like subdomains, respectively. (**E**) SEM of the complex morphology that emerges at Λ⪆ 70 nm.

The azimuthal angle of maximum SAXS intensity along the primary BCP peak yields an average alignment angle for the morphology within the combinatorial sample (χ*_c_*) that can be plotted across the template design space, as shown in Fig. 4A, where χ*_c_* = 0° when patterns align orthogonally to the template grating direction. χ*_c_* is ill-defined in the isotropic region (Λ < *L*_0_) and hence is distributed randomly there. On the other hand, for Λ > 2*L*_0_, χ*_c_* clusters near zero, which we attribute to scattering signal from the rungs of the ladder pattern. The most striking feature, however, is the negative correlation between χ*_c_* and Λ in the region where skew patterns predominate. There are two distinct populations of χ*_c_* that are responsive to the template, as discussed in the Supplementary Materials (including fig. S7).

SEMs obtained from selected locations within skew region (Λ= 60 to 80 nm), an example of which is shown in Fig. 4B, reveal dots on a hexagonal lattice, although the lattice is often rotated by ~30° from the cases near Λ = *L*_0_ in which the hexagonal dot patterns are well registered to the chemical pattern template. Furthermore, numerous lines are observed to connect these dots at angles from the direction orthogonal to the template grating, which are consistent with χ*_c_*. We infer that the observed morphology is a bilayer, depicted schematically in Fig. 4C, where vertical posts on a hexagonal lattice assemble at the template interface, while lines assemble at the air interface. For added clarity, in Fig. 4D, we mark the hypothesized posts and lines in a SEM from the skew region taken at a 45° tilt by dotted blue arrows and dashed green lines, respectively. A simple trigonometric calculation based on this bilayer model can provide a plausible upper limit for the relationship between χ*_c_* to Λ, as discussed in the Supplementary Materials. The bilayer motif appears to break down at Λ ⪆ 70 nm into a more complex morphology (Fig. 4E) with reduced dependence of χ*_c_* on Λ that is reminiscent of nonbulk morphologies previously observed for symmetric diblock copolymers on highly incommensurate chemical patterns (*34*, *35*). Note that multiple types of morphologies coexist within the skew region of the template parameter space, indicating an energetic degeneracy among them (see fig. S8). Additional tilt-view SEMs from the skew region that support our designation of the bilayer morphology are provided in the fig. S9.

We posit that the bilayer morphology and other unusual patterns observed in the skew region are a consequence of the capacity of the blend to accommodate incommensurability between Λ and *L*_0_ by locally partitioning into distinct morphological objects, or subdomains, within a morphological unit cell of area Λ × *L*_0_ (bounded vertically by the film thickness). This partitioning can be thought of as the result of highly localized enrichment of BCP chains directed by the chemical pattern template. In the absence of external directing fields, energetic penalties associated with chain stretching or compression due to chain mixing promote localized enrichment of cylindrical and lamellar BCP chains within a thin film blend of lamellar and cylindrical BCPs of nearly equivalent molecular weight, as used here, leading to the self-assembly of coexisting dot and lines patterns (*24*, *36*). On the other hand, a chemical pattern template with features nearly commensurate with the BCP domains imposes a strong directing field that can enforce the assembly of a single pattern type (i.e., lines or dots), aligned and registered with the template, which minimizes chain distortion while satisfying surface wetting constraints (*23*). As the template pitch increases, however, a single pattern type (i.e., only lines or only dots) cannot accommodate the resulting incommensurability between template and domain spacing. For templates in the range of *L*_0_ < Λ < 2*L*_0_, the space between grating lines is insufficient to enable distinct subdomains to coexist at their natural spacing within a single layer in registration with the template. In response, the blend may self-assemble to form a bilayer morphology with layers partitioned according to the general subdomain shape. Although subdomain shapes often change continuously with position throughout the film, we find it useful to classify subdomains as “cylinder-like” when enrichment of cylindrical BCP chains either between or within the subdomains stabilizes higher domain interfacial curvature. On the other hand, similar enrichment of lamellar BCP chains can stabilize “lamella-like” subdomains with lower domain interfacial curvature.

**Fig. 5.**
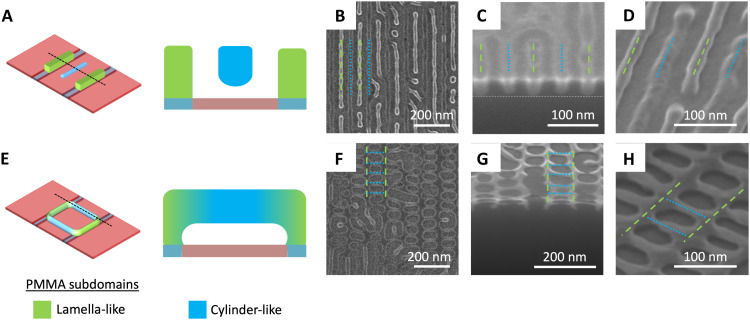
Emergent patterns in proximity to Λ ≈ 2*L*_0_. Schematic unit cell (**A**) and top-view (**B**), cross-sectional (**C**), and tilt-view (**D**) SEMs of alternating cylinder-like (i.e., enriched with PMMA-minority BCP chains) and lamella-like (i.e., enriched with symmetric BCP chains) subdomains aligned along the grating direction, which emerge in the vicinity of Λ ≈ 2*L*_0_ and larger values of *w*. Schematic unit cell (**E**) and top-view (**F**), cross-sectional (**G**), and tilt-view (**H**) SEMs of ladder structures, which emerge in the vicinity of Λ ≈ 2*L*_0_ and smaller values of *w* or in regions where Λ is slightly more or less than 2*L*_0_. Green and blue represent lamella-like and cylinder-like PMMA subdomains, respectively. Right schematics represent cross-sectional slices at the position of the dotted lines in the left schematics. Green dashed and blue dotted lines in the SEMs indicate the inferred positions of lamella-like and cylinder-like subdomains, respectively.

The patterns found in the vicinity of Λ ≈ 2*L*_0_ can again be understood as a product of the partitioning of the blend into cylinder-like and lamella-like subdomains within a unit cell; here, however, the partitioning occurs within a single layer, which is made possible by the larger spacing between template grating lines. For larger *w* at Λ ≈ 2*L*_0_ (Fig. 2, H and I), lamella-like PMMA subdomains assemble directly above the template grating to maximize favorable contact between PMMA and hydrophilic regions, while cylinder-like PMMA subdomains align horizontally and in parallel with the PMMA lamella-like subdomains, as depicted schematically in Fig. 5A. The line patterns in this area of the template space are thus composed of alternating and aligned subdomains. This configuration minimizes both enthalpic penalties due to domain/template mismatch and entropic penalties due to chain stretching while satisfying the constraint of equal volume for cylindrical and lamellar chains. Accompanying SEMs (Fig. 5, B to D) are consistent with the alternating subdomain model. Here, we mark the hypothetical lamella-like and cylinder-like subdomains by dashed green and dotted blue lines, respectively. PMMA within the nominal lamella-like subdomains above guiding lines extends across the entire film thickness, resulting in a higher degree of alumina infiltration that provides brighter contrast and more prominent alumina replicas of these subdomains in top-view (Fig. 5B) and tilted-view (Fig. 5D) SEMs, respectively. A horizontal dotted white line in the cross-sectional SEM (Fig. 5C) marks the bottom of the lamella-like subdomains, whereas the alternating cylinder-like subdomains are identifiable by their reduced depth. The apparent flat or concave surfaces at the top of the subdomains in Fig. 5C are likely an artifact from the milling process used to obtain the cross section.

As *w* decreases (Fig. 2J), the mismatched contact area between PMMA subdomains and the hydrophilic template lines introduces an enthalpic energy penalty; reducing the subdomain width, however, compresses chains within the subdomains above guiding lines and stretches chains within the alternating subdomains. The blend can resolve this dilemma by orienting the cylinder-like PMMA subdomains orthogonally to the template grating line direction to minimize chain distortion, resulting in the ladder morphology depicted schematically in Fig. 5E and shown in a representative top-view SEM in Fig. 5F. Under this hypothesis, lamella-like PMMA subdomains form the ladder rails, while cylinder-like PMMA subdomains form the rungs. For added clarity, we again mark the hypothetical lamella-like and cylinder-like subdomains by dashed green and dotted blue lines, respectively. Cross-sectional and tilt-view SEMs [Fig. 5 (G and H, respectively)] demonstrate that the ladder morphology contacts the substrate along the ladder rails, which are connected to each other by suspended rungs, consistent with the proposed mechanism for ladder self-assembly.

The orientation of the subdomains orthogonally to the template guiding lines to minimize chain stretching also explains why ladder patterns dominate at Λ slightly more or less than 2*L*_0_. Lamella-like PMMA assembles on top of the template lines to minimize mismatched contact area, while the orthogonal horizontal cylinder-like PMMA subdomains fill the space between template lines without incurring an enthalpic penalty. Measurements of the spacing between rungs from SEMs at Λ values ranging from ~90 to 130 nm maintain a consistent value of 54 ± 2 nm. The equivalence of the rung spacing with the equilibrium domain spacing for the overall blend across a large range of Λ supports the proposed mechanism for ladder assembly. Additional SEMs provided in figs. S10 and S11, including cross sections obtained from milling by a focused ion beam, reinforce the conclusions regarding the three-dimensional morphologies observed in the vicinity of Λ ≈ 2*L*_0_.

**Fig. 6.**
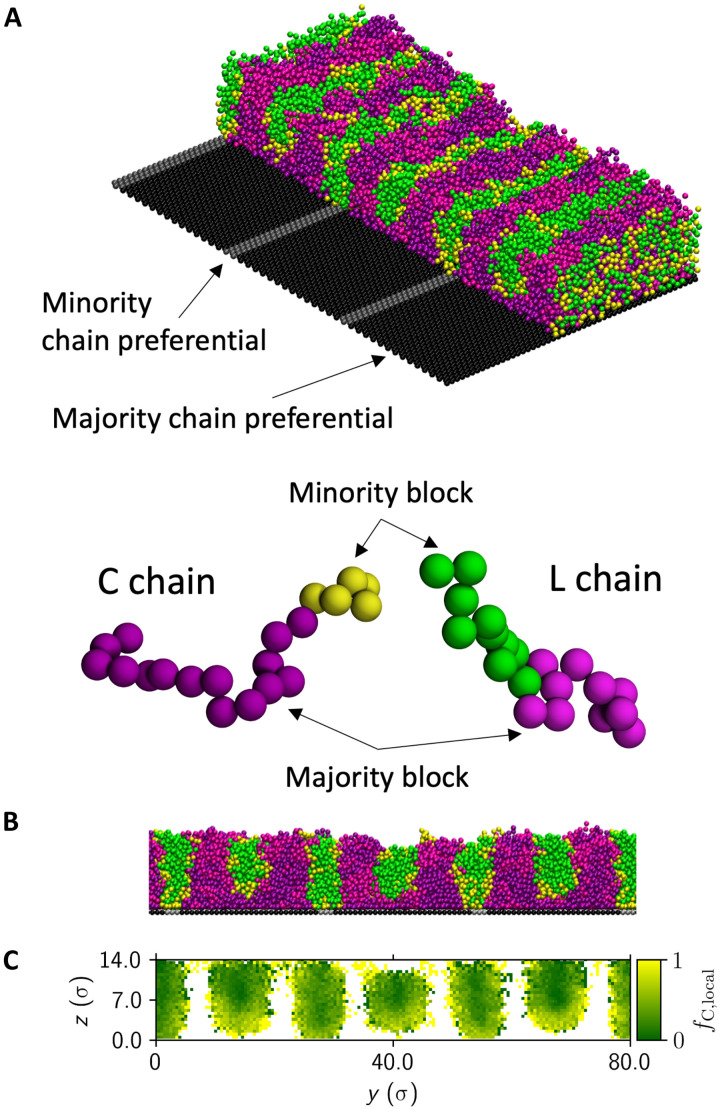
Exemplar coarse-grained MD simulation result for a cylindrical/lamellar BCP blend on a chemical grating template with Λ ≈ 2*L*_0_ and *w*Λ = 0.100. Black substrate beads are preferential for majority BCP chains, analogous to PS brush–grafted regions, while gray substrate beads are preferential for minority BCP chains, analogous to etched grating lines. Symmetric L chains consist of blocks colored green and magenta, while the minority and majority blocks in asymmetric C chains are colored yellow and purple, respectively. Minority beads in the different chain types are chemically identical and are colored differently only for visualization purposes (similarly for majority beads). A perspective view of the result with a portion of the film removed to reveal the underlying chemical pattern (**A**) and a cross-sectional view taken orthogonally to the chemical grating direction (**B**) reveal a remarkable agreement with the experimentally observed alternating morphology (in the vicinity of Fig. 2H). The local fraction of cylindrical minority BCP chains (*f*_*C*, local_) plotted in (**C**) reveals enrichment of asymmetric (cylindrical) chains near the substrate between grating lines. This local redistribution of chains stabilizes the assembly of aligned cylinder-like subdomains. Here, “*y*” is the direction perpendicular to the gratings; “*z*” is the substrate normal direction, and the unit (σ) is the bead size parameter.

Using coarse-grained molecular dynamics (MD) simulations, we have previously shown that highly localized chain redistribution and enrichment at the scale of single morphological objects in thin film blends of cylindrical and lamellar BCPs stabilizes coexistence phases (i.e., dots and lines) (*36*). We therefore used MD simulations to investigate the assembly behavior of a blend of “L” (symmetric) and “C” (asymmetric) BCP chains on a wide range of chemical patterns analogous to those used experimentally. Simulation details are provided in Materials and Methods. An exemplar MD simulation result is shown in perspective view in Fig. 6A, with a portion of the film removed to reveal the underlying chemical pattern, and in a cross-sectional view along a slice taken orthogonally to the chemical grating direction in Fig. 6B. At these template dimensions (Λ ≍ 2*L*_0_ and *w*/Λ = 0.100), corresponding to the template region in the vicinity of Fig. 2H, the agreement with the experimentally observed alternating morphology is remarkable. Simulated minority subdomains (PMMA in the experiment) contact the substrate at the location of the grating lines, whereas the alternating minority subdomains are separated from the brush-grafted substrate regions by majority block chains.

To elucidate the role of localized chain enrichment in the self-assembly of the alternating morphology, the average molecular fraction of C minority block chains (*f*_C, local_) along the grating direction is plotted in Fig. 6C versus the substrate normal direction (*z*) and the direction perpendicular to the gratings (*y*), where the color code between C and L minority species matches the chain schematic in Fig. 6A. Notably, *f*_C, local_ increases near the substrate within alternating subdomains not above grating lines, signaling a localized enrichment of C BCP chains that accommodates the higher interfacial curvature required to separate the minority subdomain from the unfavorable brush-grafted substrate. Our MD simulations therefore strongly support our hypothesis that localized cylindrical or lamellar chain enrichment is essential to the assembly of the previously undiscovered morphologies described in this report.

As shown in the Supplementary Materials, simulations performed on chemical patterns in which *w* is reduced or Λ is shifted away from ~2*L*_0_ reveal a self-assembled morphology that bears a remarkable resemblance to the proposed bilayer structure for skew patterns and occurs in the same general regions of the template parameter space (figs. S12 and S13). While less common, hints of the ladder morphology are also observed in simulations across these same regions, as evidenced in figs. S12 and S14. Moreover, alternating morphologies become more common as film thickness is reduced (figs. S15 and S16), while a tendency toward defective, ladder-like morphologies is apparent when film thickness is increased (fig. S17). This suggests that film thickness may play an important role in the presence or absence of specific emergent morphologies formed through subdomain partitioning.

A high level of defectivity is generally observed in self-assembled patterns across the entire template. In part, this is a natural outcome of self-assembly in polymer blends, which can stabilize template-programmable defects (*22*). As the objective of this work is to deploy the synergistic combination of combinatorial sampling and autonomous characterization for morphological discovery, we did not endeavor to optimize our templates for the defect-free assembly of any particular morphology. Nevertheless, our results highlight key trends that may lead to formalized template design rules for morphological selection. As an example, both experiment and simulations show that aligned and alternating subdomains are largely constrained to larger values of *w* near Λ ≈ 2*L*_0_ (see Figs. 2 and 3B). Consideration of past research on BCP DSA using chemical patterns (*31*, *37*) suggests that this alternating morphology would be most strongly selected for when *w* ≈ 0.5*L*_0_, as this would minimize enthalpic penalties incurred at the interface between the template and lamella-like subdomains. Meanwhile, the ladder morphology is clearly dominant at Λ > 2*L*_0_ (Fig. 3E), a region of the template design space where it does not compete with the skew morphology. Considering the morphology transition from ladder to alternating lines with increasing *w* at Λ ≈ 2*L*_0_, the ladder morphology may be most strongly selected for when Λ > 2*L*_0_ and *w* ≪ 0.5*L*_0_.

## DISCUSSION

We have characterized complex three-dimensional morphologies that emerge via self-assembly of BCP blend thin films in response to underlying chemical pattern templates. By autonomously mapping a combinatorial sample using synchrotron x-ray scattering, we have substantially expanded the explorable parameter space compared to previous experiments while simultaneously providing multifaceted morphology descriptors, all without immediate human intervention. The autonomously derived results guided subsequent electron microscopy characterization, enabling the judiciously targeted selection of regions to image for interpreting the assembly behavior. This investigation revealed the emergence of previously unknown morphologies such as a bilayer structure, alternating gratings, and a ladder morphology; it also helped distill the general principle of subdomain partitioning that can be used to predict, interpret, and design patterns by the DSA of BCP blends, thereby adding a valuable mechanism to engineer hierarchical self-assembled morphologies with precise registry control. While our results are germane to selective DSA using BCP blends, the general approach to autonomously characterizing large parameter spaces demonstrated here provides a wealth of crucial information to understand complex assembly behavior while freeing up human experimenters to focus on interpretation and hypothesis formulation.

## MATERIALS AND METHODS

### Materials

Hydroxyl-terminated PS “brush” (PS-OH, number average molar mass (M_n_) = 10.5 kg mol^−1^, polydispersity index (PDI) = 1.06), lamellae-forming PS-*b*-PMMA (M_n_ = 52-*b*-52 kg mol^−1^, PDI = 1.09), and cylinder-forming PS-*b*-PMMA (M_n_ = 64-*b*-35 kg mol^−1^, PDI = 1.09) were obtained from Polymer Source and used as received. The PS brush was dissolved in propylene glycol monomethyl ether acetate (PGMEA) at a concentration of 1% by weight, while the BCPs were dissolved in toluene at a concentration of 1% by weight. BCP solutions were prepared by combining the two pure BCP solutions in a 1:1 weight ratio. Polished silicon wafers that were 200 μm thick were purchased from University Wafer.

### Sample fabrication

After cleaning by O_2_ plasma treatment, the PS brush was grafted to a silicon wafer coupon with dimensions of ~2 cm by ~3 cm that was spin-casting a film of it at 1500 rpm and annealing on a hot plate for 5 min at 250°C under continuous nitrogen purging to chemically graft the brush to the substrate. Ungrafted brush was removed by spin-rinsing in PGMEA at 3000 rpm. PMMA electron beam resist (950 K) was spin-coated to a thickness of ∼50 nm and baked on a hot plate at 180°C for 3 min. Line/space grating patterns were exposed in a JEOL JBX6300-FS electron beam lithography tool using 1-nA beam current with doses ranging from 1200 to 2080 μC cm^−2^. After exposure, the samples were developed in room temperature methyl isobutyl ketone:isopropyl alcohol (1:3) for 60 s and rinsed in isopropyl alcohol. Exposed grating patterns were transferred to the PS brush by oxygen plasma etching (Trion Phantom) RIE tool using 10 standard cubic centimeters per minute (sccm) O_2_ at 50 mtorr, with 15-W radio frequency power for ∼40 s. The remaining PMMA was removed by soaking in toluene at 60°C for 15 min, with the final 10 min in an ultrasonic bath, followed by soaking in N-Methyl-2-pyrrolidone at 180°C for 10 min in an ultrasonic bath, and lastly in room temperature methanol for 5 min in an ultrasonic bath. Grating linewidths were simulated across the entire combinatorial array using the BEAMER software package (GenISys). A point spread function for PMMA on silicon was applied on the grating pattern in the electron beam simulation module, looping over a range of grating pitches and electron beam doses that matched the exposure conditions. The BEAMER metrology module was used to extract linewidths at each array position.

The BCP blend film was subsequently spin-casted onto the chemical pattern from toluene solution at 2000 rpm and thermally annealed in a vacuum oven at 220°C for ~18 hours. After annealing, alumina replicas of the three-dimensional PMMA domain structure were synthesized through vapor phase infiltration as described previously (*38*) to enhance contrast for electron microscopy and x-ray scattering. Briefly, the sample was placed into a commercial atomic layer deposition tool (Cambridge Ultratech Savannah S100) with a base pressure <1 torr and exposed to four cycles of exposure to trimethylaluminum and water vapor (100 s each) at 85°C. The polymer was then removed by oxygen plasma ashing at 20 W and ~100 mtorr (March Plasma CS1701F) to reveal the alumina replicas.

### X-ray scattering characterization

Transmission SAXS experiments were conducted at the SMI (12-ID) beamline at the NSLS-II at Brookhaven National Laboratory. The beamline x-ray energy was set to 16.1 keV (corresponding to x-ray wavelength of 0.7701 Å). SAXS data were collected on a photon-counting pixel array detector (Dectris PILATUS 1M) with a pixel size of 172 μm. Data were converted to reciprocal space (*q*) using the calibrated sample-detector distance (8.3020 m). Data were analyzed using the SciAnalysis software package, which computes a variety of signals for each experimental detector image. We computed the isotropic one-dimensional scattering curve *I*(*q*) based on the azimuthal average of the full SAXS pattern, as well as the sectored one-dimensional curve for a variety of angles (from 0° to 90° relative to the grating line direction). For each pattern, the position, width, and height of the scattering peak were quantified by fitting to a Gaussian peak with a linear background. The scattering intensity in the azimuthal direction (χ_c_), at the *q* value of the BCP first-order peak (i.e., *q*_0_ ~ 0.0117 Å; which corresponds to real space distances of *d*_0_ ~ 54 nm), was extracted. From this *I*(χ_c_) data, we fit a circularly wrapped orientation distribution function to quantify the alignment direction (*39*–*42*)

$$I(\chi_{c})=\frac{{\left(1+\eta \right)}^{2}-4\eta }{{\left(1+\eta \right)}^{2}-4\eta \cos^{2}\chi_{c}}$$ (2)

where η quantifies the anisotropy. We also fit these data to a weighted sum of orientation functions for the different morphology populations; the relative weights thereby provide estimates of population contributions to the measured scattering intensity. We define the “line” population as a *I*(χ_c_) distribution with twofold symmetry and aligned along χ_c_ = 90°, the “hex” population as sixfold symmetric aligned along 30°, the “skew” population as fourfold symmetric aligned along 45°, and the ladder population as twofold symmetric aligned along 0°.

For autonomous characterization, we fed the selected analysis signals into the gpCAM (*29*) software, which implements GP modeling for arbitrary signals across arbitrarily dimensioned parameter spaces (*25*–*28*). The underlying kernel was anisotropic (*28*), accounting for differences in the correlations along the *x_c_* (Λ) and *y_c_* (*w*) directions. The presented maps of analysis signals were generated using a GP model, with hyperparameters trained on the full dataset. Thus, they represent an interpolated map that takes into account the experimental data, its corresponding uncertainty, and the learned correlation behavior through the parameter space. Autonomous control was implemented using an objective function that included a term proportional to the surrogate model uncertainty and a term proportional to the data signals; we used the scattering peak intensities (in the 30°, 60°, and 90° azimuthal directions) as the signals of interest.

### Electron microscopy

Top-down imaging was performed using a Hitachi S-4800 scanning electron microscope at 10-kV accelerating voltage. Cross-sectional and tilt-view SEMs were acquired using a FEI Helios scanning electron microscope at 5-kV accelerating voltage.

### Coarse-grained MD simulations

MD simulated films were made from linear A-*b*-B BCP chains described by a coarse-grained, Kremer-Grest bead-spring model (*36*, *43*–*45*). Asymmetric (cylinder-forming) “C” and symmetric (lamellae-forming) “L” chains are the same length (20 beads) and are differentiated by their A block fraction: The A block fraction is 0.25 and 0.5 for the asymmetric and symmetric BCPs, respectively. Each bead has unit mass (1.0*m*) for all types, and the interactions between BCP chain beads consist of bonded and nonbonded potentials. Parameters *m*, σ, ϵ, and τ are units for mass, distance, energy, and time, respectively, and τ = σ(*m*/ϵ)^1/2^. The bonded interactions between every pair of adjacent chain beads are governed by a finitely extensible nonlinear elastic (FENE) potential as described in Eq. 3, which is a sum of attractive (first term) and repulsive (second term) potentials, resulting in a wall-like potential with an equilibrium length

$$U_{\mathrm{FENE}}(r)=-0.5k{R_{0}}^{2}\ln\left(1-\frac{r^{2}}{{R_{0}}^{2}}\right)+4\epsilon_{ij}\left[{\left(\frac{\sigma_{ij}}{r}\right)}^{12}-{\left(\frac{\sigma_{ij}}{r}\right)}^{6}+\frac{1}{4}\right]$$ (3)

where *r* is the distance between beads, *k* is a spring constant, *R*_0_ is a maximum length, ϵ*_ij_* is the strength of interaction between a bead of type *i* and a bead of type *j*, and σ*_ij_* is the finite distance at which the interbead potential is zero. The second term is cut off and shifted to zero energy at *r_c_* of $\sqrt[{6}]{2}$, and thus is purely repulsive. The nonbonded interactions are modeled using a Lennard-Jones (LJ) potential as described in Eq. 4 and apply to every pair of beads except covalently bonded ones. ϵ*_ij_* and σ*_ij_* share the same definitions as in Eq. 3

$$U_{\mathrm{LJ}}(r)=\{\begin{array}{c} 4\epsilon_{ij}\left[{\left(\frac{\sigma_{ij}}{r}\right)}^{12}-{\left(\frac{\sigma_{ij}}{r}\right)}^{6}\right],r\leq r_{c}\\ 0,r>r_{c} \end{array}$$ (4)

The LJ potential is cut off and shifted to zero at a *r_c_* of 2.5σ, due to which the nonbonded interactions include attractive interactions. We set potential parameters so that the self-cohesion of A block can be slightly stronger than that of B block as given in Table 1, which mirrors a BCP such as PS-*b*-PMMA where PMMA and PS are A and B blocks, respectively.

**Table 1.** Parameters used for FENE and LJ potentials.

Parameter	Value
*k*	30ϵ/σ^2^
*R*_0_	1.5σ
*r_c_* (FENE)	$\sqrt[{6}]{2}\sigma$
*r_c_* (LJ)	2.5σ
σ_AA_ = σ_BB_ = σ_AB_ = σ_SA_ = σ_SB_	1.0σ
ϵ_AA_	1.01ϵ
ϵ_AB_ = ϵ_BA_	0.50ϵ
ϵ_BB_	0.99ϵ

We used the same number of symmetric and asymmetric BCPs to construct each film in the simulation box with a chemical pattern at the bottom surface, depicted in fig. S18. *L_y_*was set to have three to four pitches in the simulation box, and then, *L_x_* was set to *L_y_*/1.618 using the golden ratio. *L_z_* is much larger than the film thickness to induce the film-air (vacuum) interface. Periodic boundary conditions were imposed in the *x* and *y* axis so that the model can represent thin films. Each film was equilibrated (annealed) up to 0.3 × 10^6^ τ with a time step of 0.006τ at *T* = 1.2ϵ/*k*_B_ using the NVT ensemble with a Nosé-Hoover thermostat. The chemical patterned substrate mirrors the experimental substrate; thin stripes of width *w* constructed from fixed S_A_ beads (preferential to the A block) are separated from each other at a pitch Λ by regions on the substrate constructed from fixed S_B_ beads, where S_A_ and S_B_ beads share the same interaction strengths (ϵ*_ij_*) as A and B beads, respectively. Λ varied from 18σ to 34σ, and the duty cycle (*w*/Λ) varied from 0.0 to 0.2. The BCP films were initially disordered with no phase separation and they self-assemble into morphologies during equilibration. The resulting films are about 12σ thick, and the domain spacing is about 13σ.

All simulations were carried out on the Institutional Cluster of the Scientific Data and Computing Center at Brookhaven National Laboratory using the LAMMPS simulation package made available by Sandia National Laboratories (*46*). Molecular snapshots were captured using the VMD software package (*47*). Different colors were used for symmetric and asymmetric BCPs to aid visualization: Yellow and green for A beads of C and L BCPs, respectively, and purple and magenta for their respective B beads. The chemical pattern B and A beads were colored black and silver, respectively. To quantify the distribution of cylindrical BCP chains in the resultant morphology, we defined the metric *f*_C, local_ as the ratio of the number of C chain beads to the total number of beads.

## Notes

View/request a protocol for this paper from *Bio-protocol*.
