# Supplementary material for: Autonomous discovery of emergent morphologies in directed self-assembly of block copolymer blends
Source: Sci Adv. 2023 Jan 13;9(2):eadd3687. doi: 10.1126/sciadv.add3687 (PMC9839324; doi:10.1126/sciadv.add3687)
Supplement: Supplementary file 1 — Supplementary Text Figs. S1 to S18 [file sciadv.add3687_sm.pdf]

Science Advances
AAAS

Supplementary Materials for

Autonomous discovery of emergent morphologies in directed self-assembly of block copolymer blends

Gregory S. Doerk *et al.*

Corresponding author: Gregory S. Doerk, gdoerk@bnl.gov; Kevin G. Yager, kyager@bnl.gov

Sci. Adv. **9**, eadd3687 (2023)
DOI: 10.1126/sciadv.add3687

This PDF file includes:

Supplementary Text
Figs. S1 to S18

Trigonometric model for the SAXS orientation angle within the skew bilayer region

For templates in the range of $L_0 < \Lambda < 2L_0$, the space between grating lines is insufficient to enable both lamellae and cylinders to coexist at their natural spacing within a single layer in registration with the template. As noted in the main text, we infer that subdomain partitioning in the skew region in which χ_c is more responsive to the template (first population) takes the form of a bilayer, with vertical cylinder-like subdomains on a hexagonal lattice at the template interface and vertical lamellae-like subdomains at the air interface. At $\Lambda > L_0$, the hexagonal lattice can rotate by 30° to minimize chain stretching. Further increases in Λ will stretch this hexagonal lattice orthogonally to the template grating, while compressing it along the direction parallel to the grating. The lattice vector initially 60° from the chemical grating normal at $\Lambda = L_0$ skews to smaller angles such that it intersects with grating lines while preserving a constant L_0 spacing between cylinder-like subdomains; lamellae-like subdomains then align above the cylinder-like subdomains along these vectors as they present the shortest distance between cylinders. In this bilayer model, χ_c can be calculated analytically by treating it as the angle of this vector (cylinder-like subdomains spaced apart by L_0 and aligned overlying lamellae-like subdomains) with respect to the grating normal, whereby χ_c is quantitatively related to pitch by $\chi_c = \cos^{-1}(\Lambda/2L_0)$, as shown in Figure S7a.

This model (solid line in Figure S7b) falls along the upper edge of the data for the first population. The bilayer motif appears to break down at $\mathit{\Lambda} \geq$ ~70 nm into a more complex morphology (Figure 4e in the main text and Figure S8) with a reduced dependence of χ_c on Λ (second population in Figure S7b). This structure is reminiscent of non-bulk morphologies previously observed for symmetric diblock copolymers on incommensurate chemical patterns (Reference 34 in the main text). Both simulation and experiment indicate domains in these morphologies orient in the substrate plane at large angles with respect to the grating direction, but not necessarily orthogonally. This occurs when incommensurability between domain and template spacing is maximized, that is when $\mathit{\Lambda}/L_0 \sim n + 0.5$, where n is an integer (Reference 35 in the main text). Similar behavior may account for the values of χ_c observed the second population in Figure S7b, as it is most prevalent when $\mathit{\Lambda}/L_0 \approx 1.3 - 1.7$. In general, the pattern motifs shown in Figures 4b and 4e of the main text are both present for $\mathit{\Lambda} \geq$ ~70 nm (see SEMs in Figure S8), along with a growing prevalence of the ladder morphology. Since χ_c is obtained through SAXS measurements across ~40 μm^2 areas, averaging of χ_c from both pattern motifs is expected to reduce its value in comparison to the predictions of this simple bilayer model. The model therefore presents a plausible upper limit for the expected dependence of χ_c on $\mathit{\Lambda}$ for the first population.

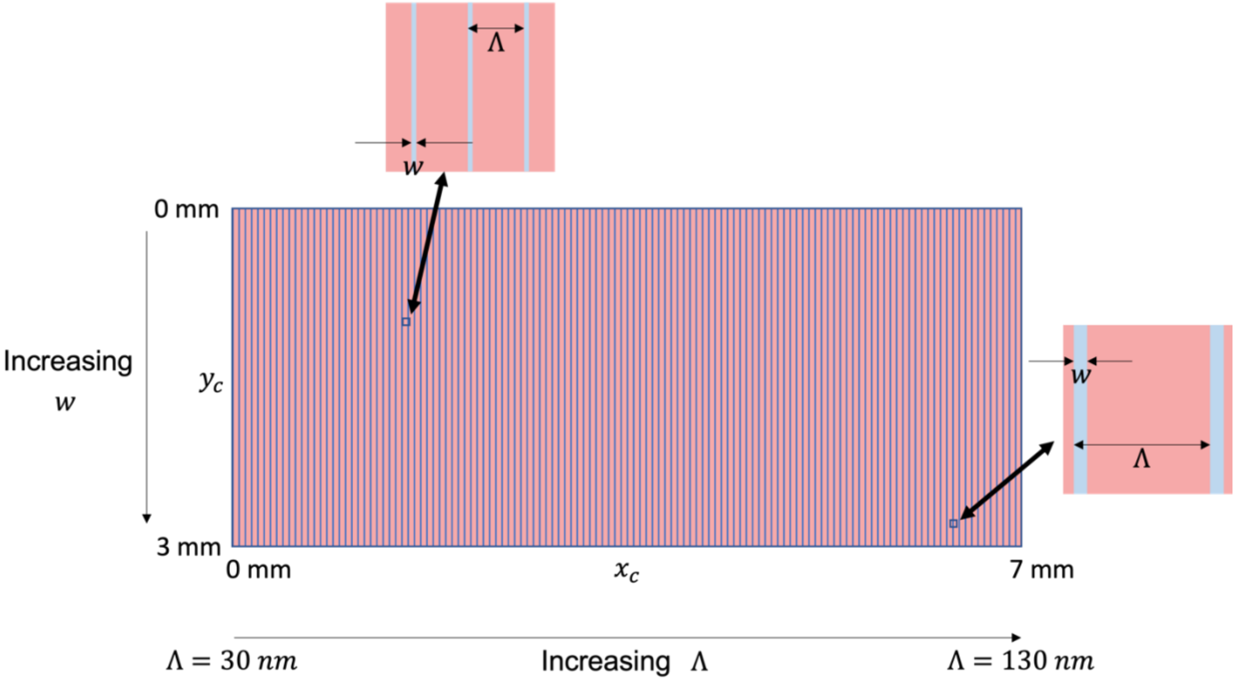

Fig. S1. Top view schematic of the combinatorial array. Insets provide schematic exemplar grating patterns with hydrophilic lines (light blue) of width *w* on a hydrophobic polystyrene brush field (light red), with a grating period of Λ. Λ increases from 30 to 130 nm across a range of x_c from 0 to 7 mm, while *w* increases across a range of y_c from 0 to 3 mm.

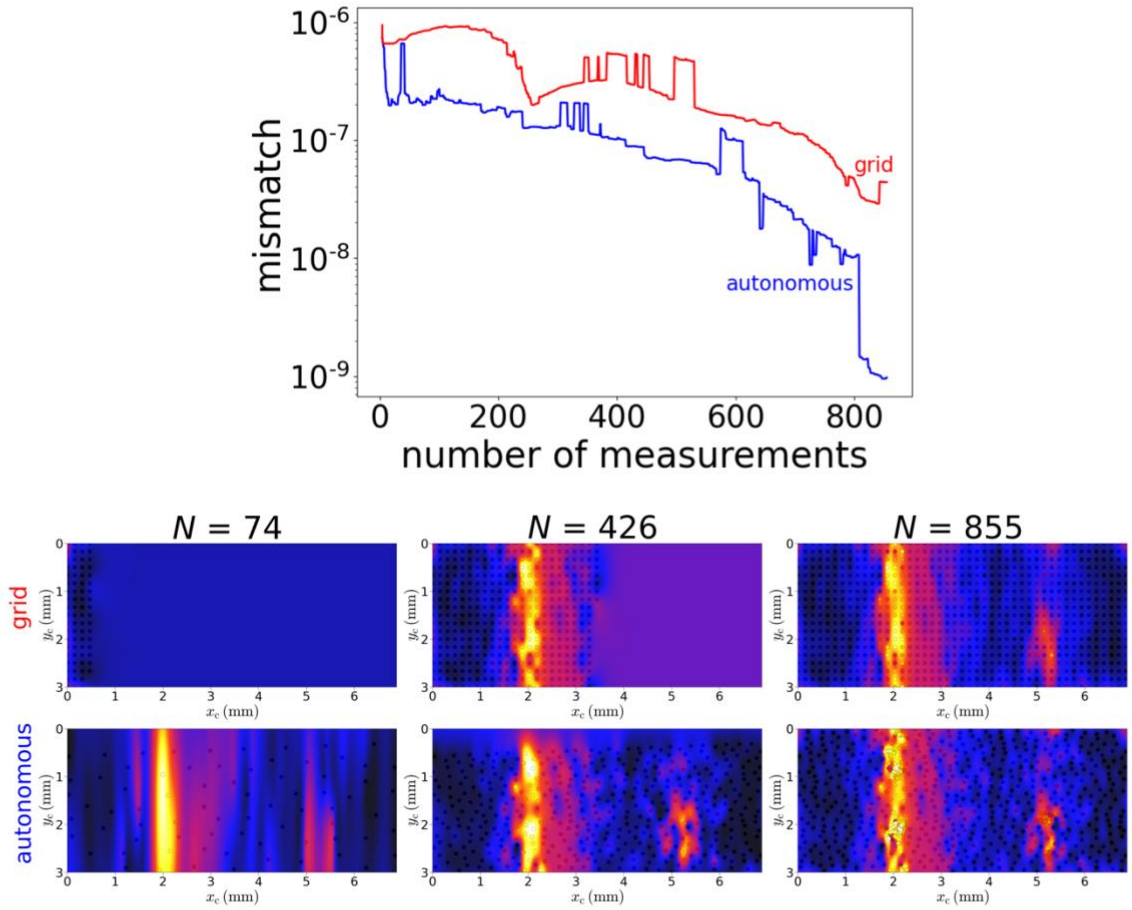

Fig. S2. **Comparison of model error, as a function of number of measurements (*N*), for the autonomous method and a simulated grid scan.** The (blue curve) autonomous method described in the main text (which selects points based on minimizing model error), and (red curve) a synthetic simulation of a straightforward grid scan. The grid scan is 855 points evenly-spaced over the scan region (19 rows and 45 columns). For each point-selection method, at each iteration a GP model is recomputed; the reported mismatch is the sum-of-squares difference between this model and a presumed correct model that is generated based on GP interpolation of the complete dataset (870 points). The grid scan simulation draws measurements from this ground truth model. A minimum filter (width 5 points) was applied to the curves to minimize the spurious jumps in the curves (which arise from changes in the optimized GP hyperparameters). The autonomous method out-performs the grid scan for a variety of reasons. The coarse-to-fine collection of AE means that it rapidly achieves a reasonable approximation over the entire space; by comparison a grid scan must complete before it can rely on interpolation instead of extrapolation. Even after full data collection, the autonomous method yields lower model error, since it has distributed points in a way that maximizes knowledge gain, and can identify small features of interest. One can also note that the regular spacing of a grid scan means that certain point-to-point length scales are never probed. The point distribution provided by AE, by comparison, provides a distribution of points allowing robust computation of spatial correlations across different scales.

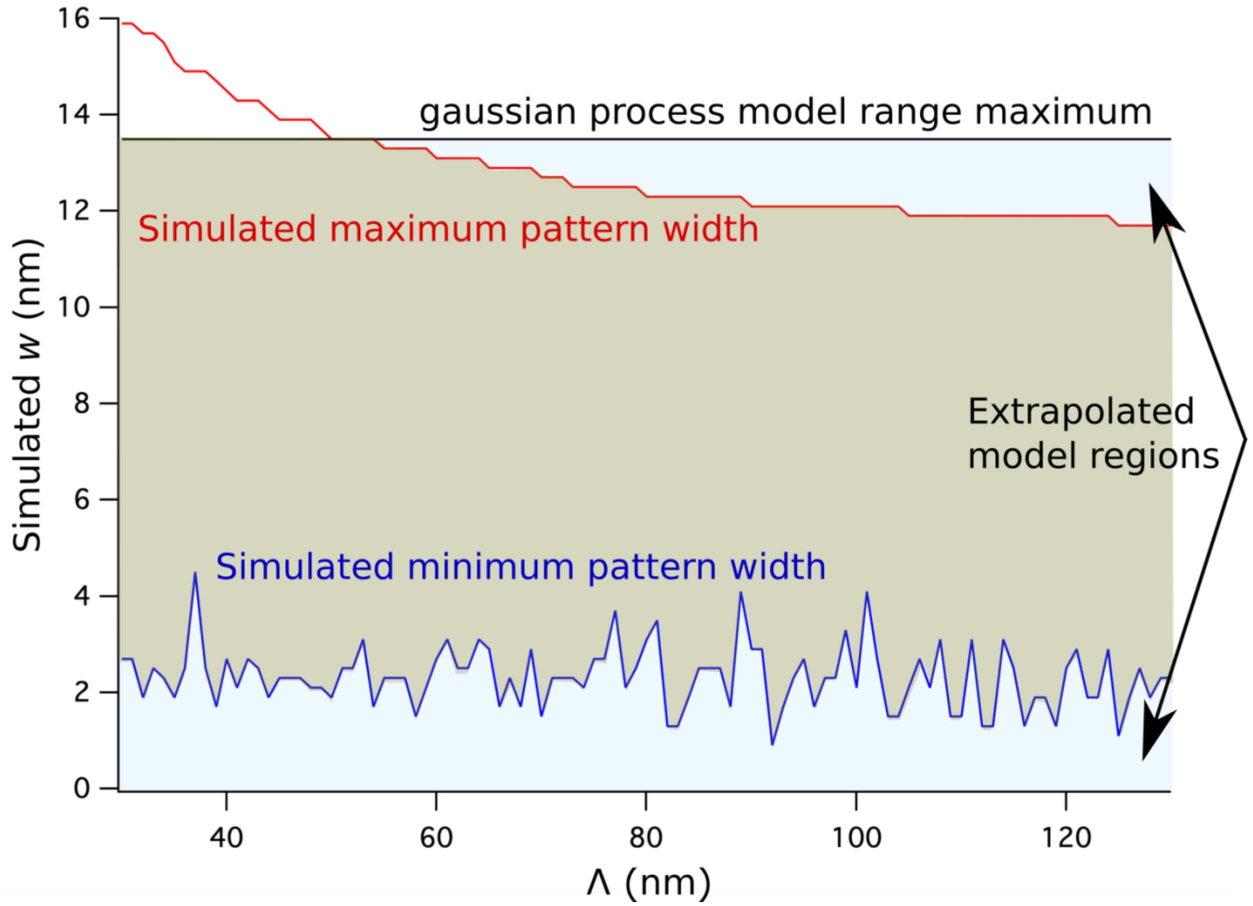

Fig. S3. Comparison with experimental parameter space with GP model range, showing the regions extrapolated by the GP model. Grating linewidths (*w*) are based on simulations using the Beamer software package, as noted in the main text.

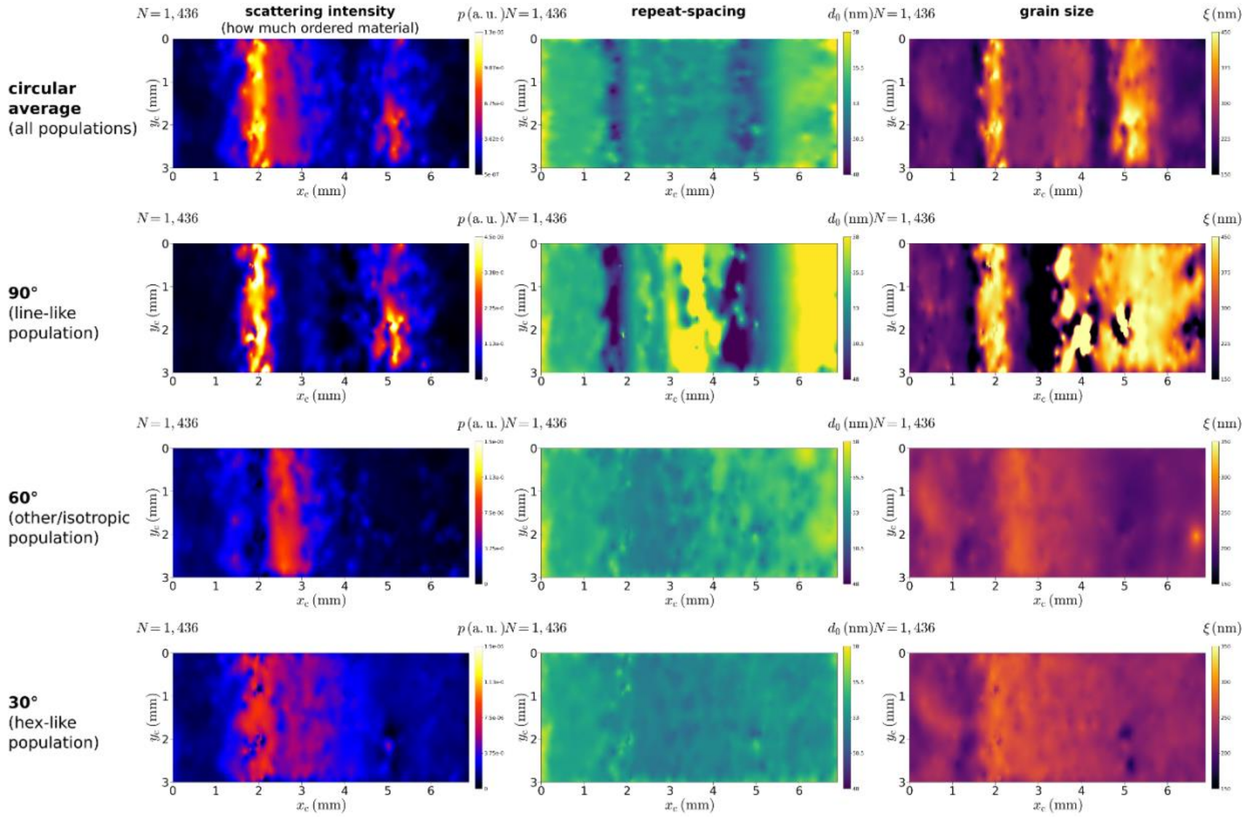

Fig. S4. Maps of various signals captured during the autonomous experiment in the combinatorial experiment coordinates (x_c, y_c). In particular, the scattering intensity (p), repeat-spacing (d_0), and grain size (ξ) are measured for four different populations. The first row is obtained by taking a circular average of the two-dimensional (2D) small-angle X-ray scattering (SAXS) image and using fitting the primary scattering peak. The subsequent rows are obtained using sector averages (at the nominal angle noted) in order to select a particular population. Analysis of the scattering data at 90° (i.e. along the grating repeat direction) provides a measure of the regions where the material is strongly aligned with the grating (e.g. line-like ordering following the template). High intensity in the 30° channel is indicative of regions of hexagonal order aligned with the grating.

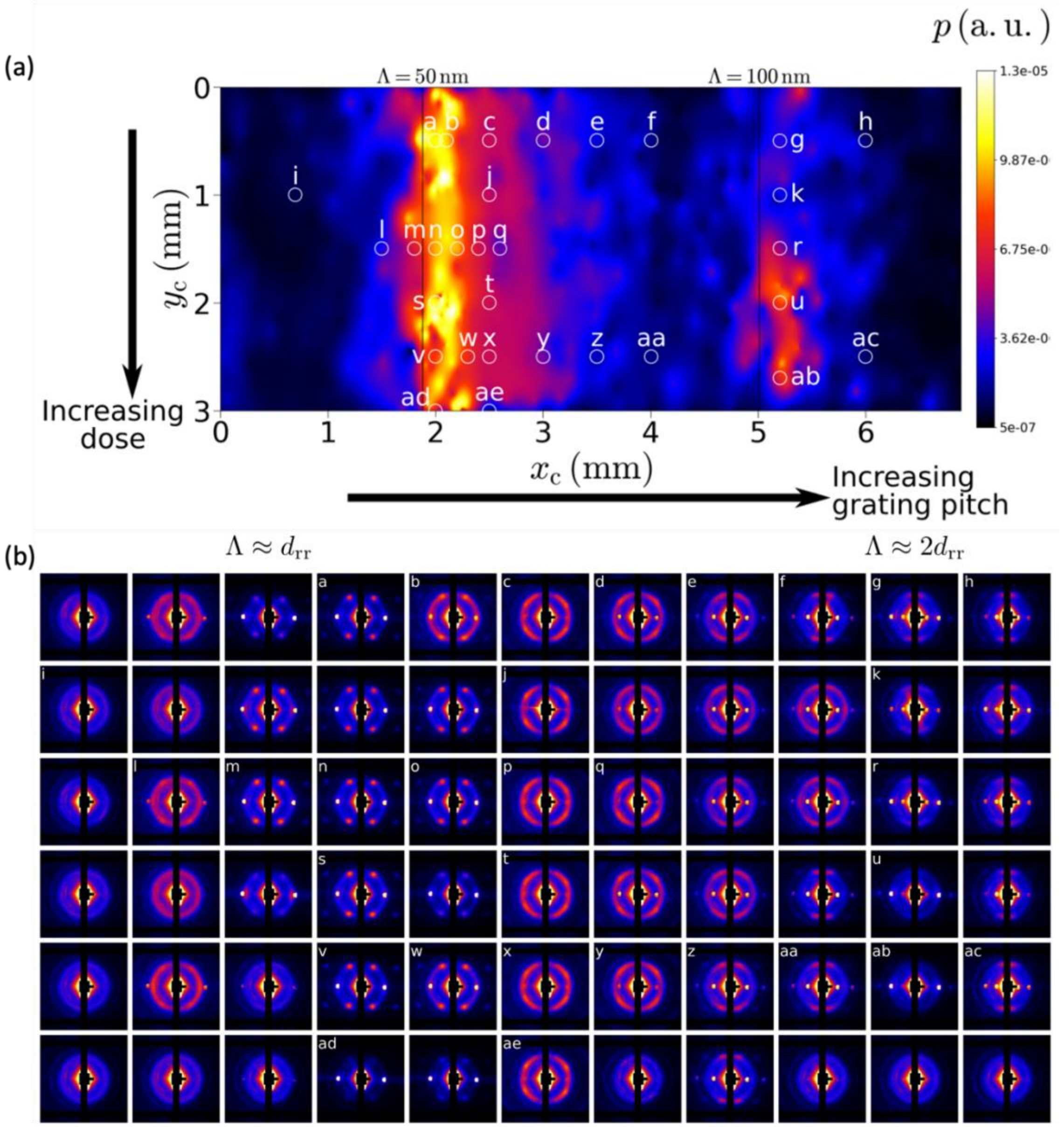

Fig. S5. Exemplar 2D SAXS patterns obtained from the autonomous experiment. (a) Map of total scattering intensity (p) obtained over the positional space of the combinatorial sample (x_c, y_c). Empty circles mark the positions of specific individual SAXS measurements. (b) Selected SAXS patterns arranged to correspond approximately to x_c and y_c in (a). Letter labels indicate SAXS patterns acquired from coordinates in the template design space depicted in (a) with the same letter labels. General trends discussed in the text are apparent. For example, from left to right the patterns transition from isotropic (featureless), to hexagonal, to skew (more intense in diagonal directions), and finally to ladder pattern motifs (high intensity at ~90 deg angle). Line type patterns become more prominent moving from top to bottom, particularly near $\Lambda \approx d_{rr}$ and $2d_{rr}$ (where d_{rr} is the self-assembled repeat spacing).

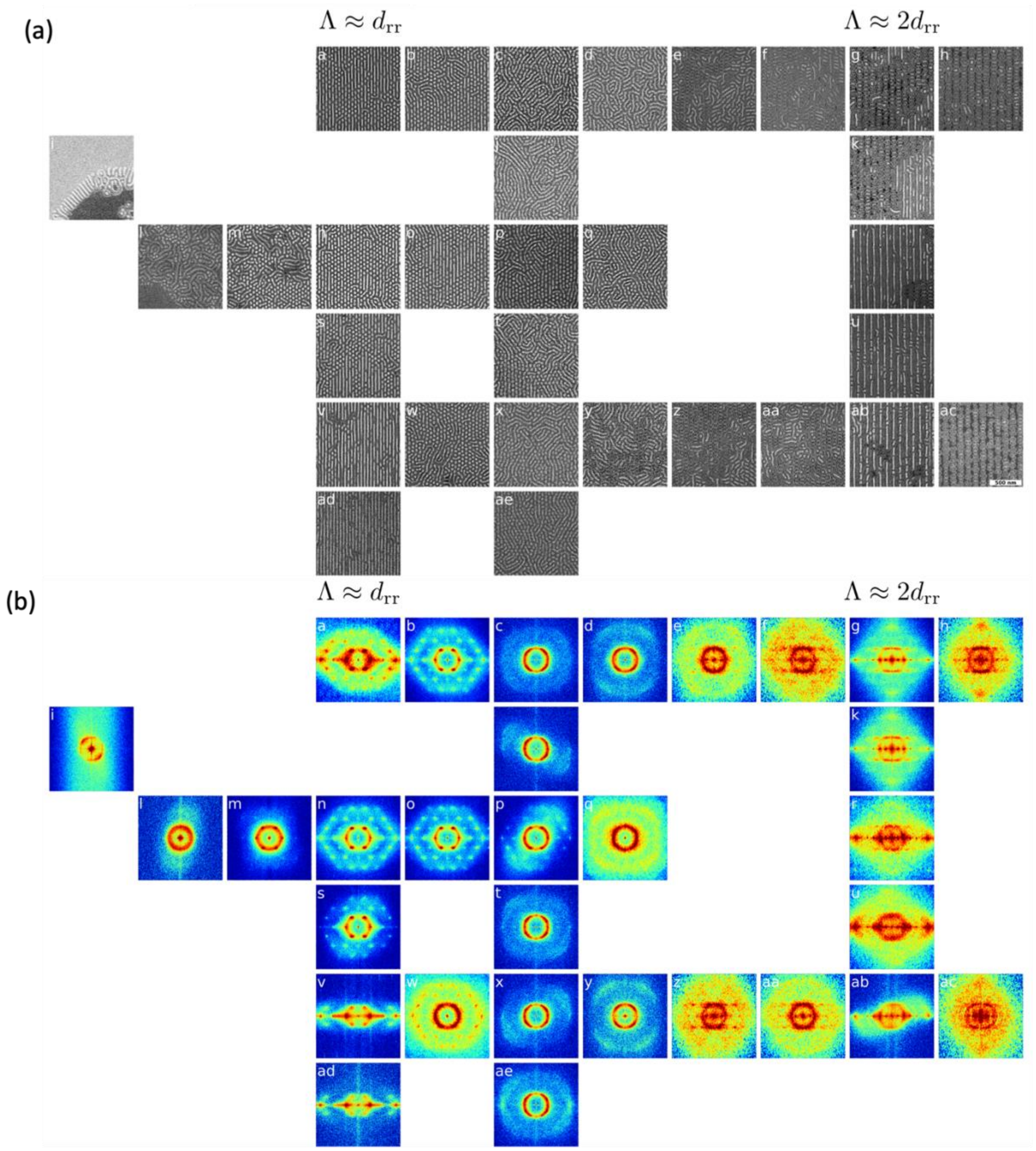

Fig. S6. Selected, manually acquired scattering electron micrographs (SEMs) acquired from the approximate positions marked by letter labels in Figure S5. (a) SEMs and (b) their corresponding Fast Fourier transforms. While necessarily sparser as a result of the manual acquisition method, the Fourier patterns qualitatively mirror the autonomously acquired SAXS patterns at the same positions, corroborating the relationships between template spacing and self-assembled pattern motifs discussed in the text.

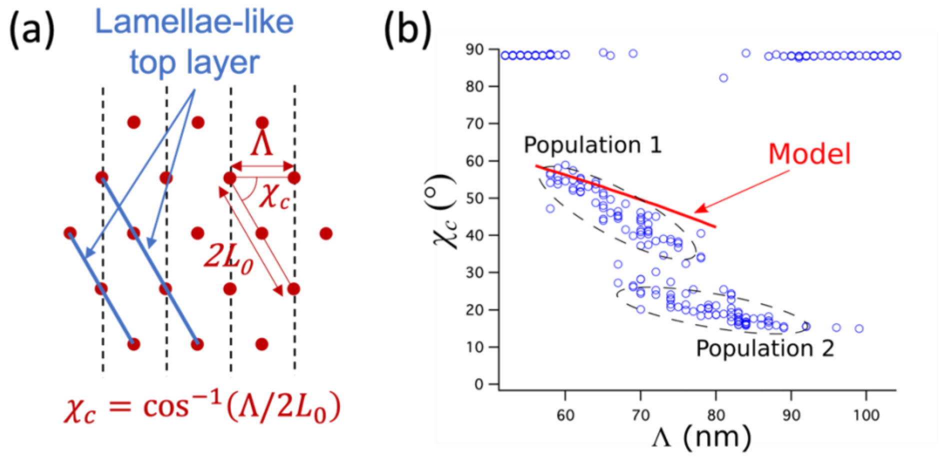

Fig. S7. Trigonometric model calculation of the SAXS orientation angle χ_c in the skew region of the template. (a) Proposed analytical model for the first χ_c population based on an assumed bilayer configuration. (b) Plot of χ_c versus Λ (for $\Lambda > L_0$), showing two populations of patterns in which χ_c is responsive to the template pitch. The red line represents the trigonometric model described previously in the Supplementary Materials.

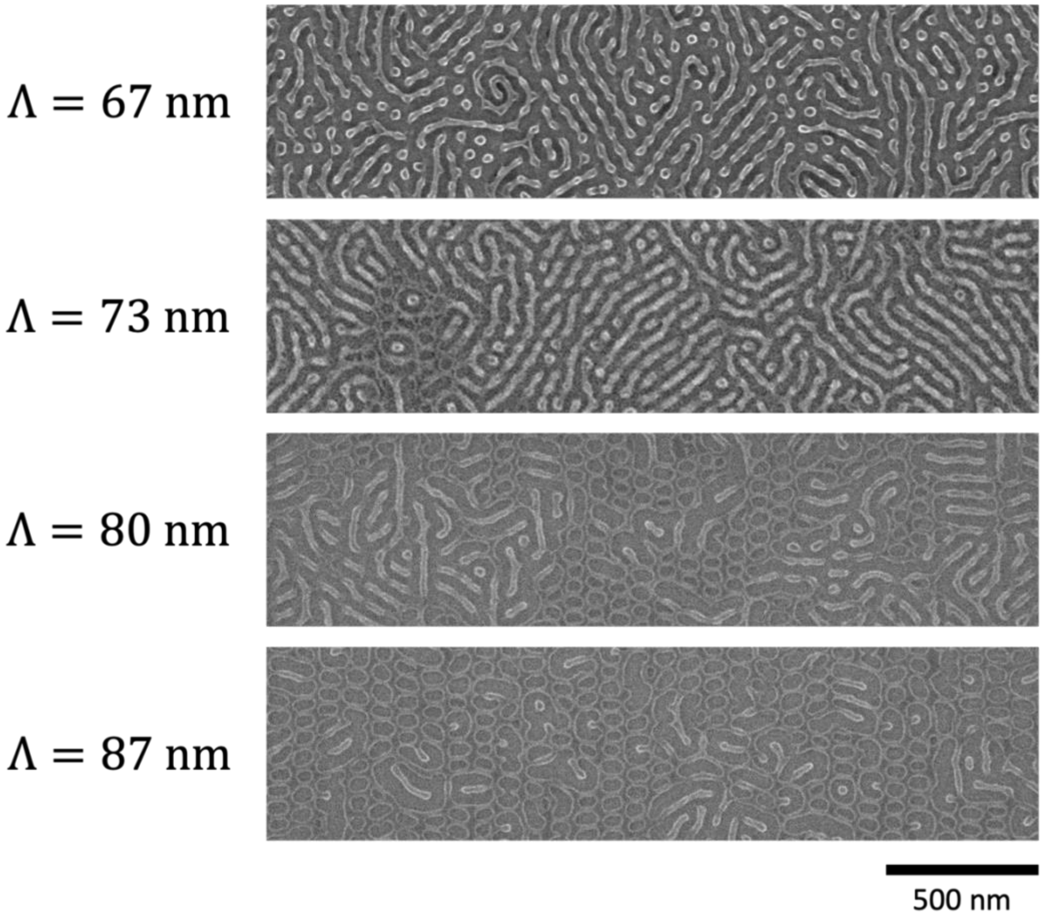

Fig. S8. Exemplary SEMs at specified values of the grating chemical pattern pitch (Λ). The emergence of the second population of self-assembled pattern alignment angles (χ_c) for Λ > ~70 nm coincides with growing prevalence of the ladder morphology. The scale bar applies to all SEMs.

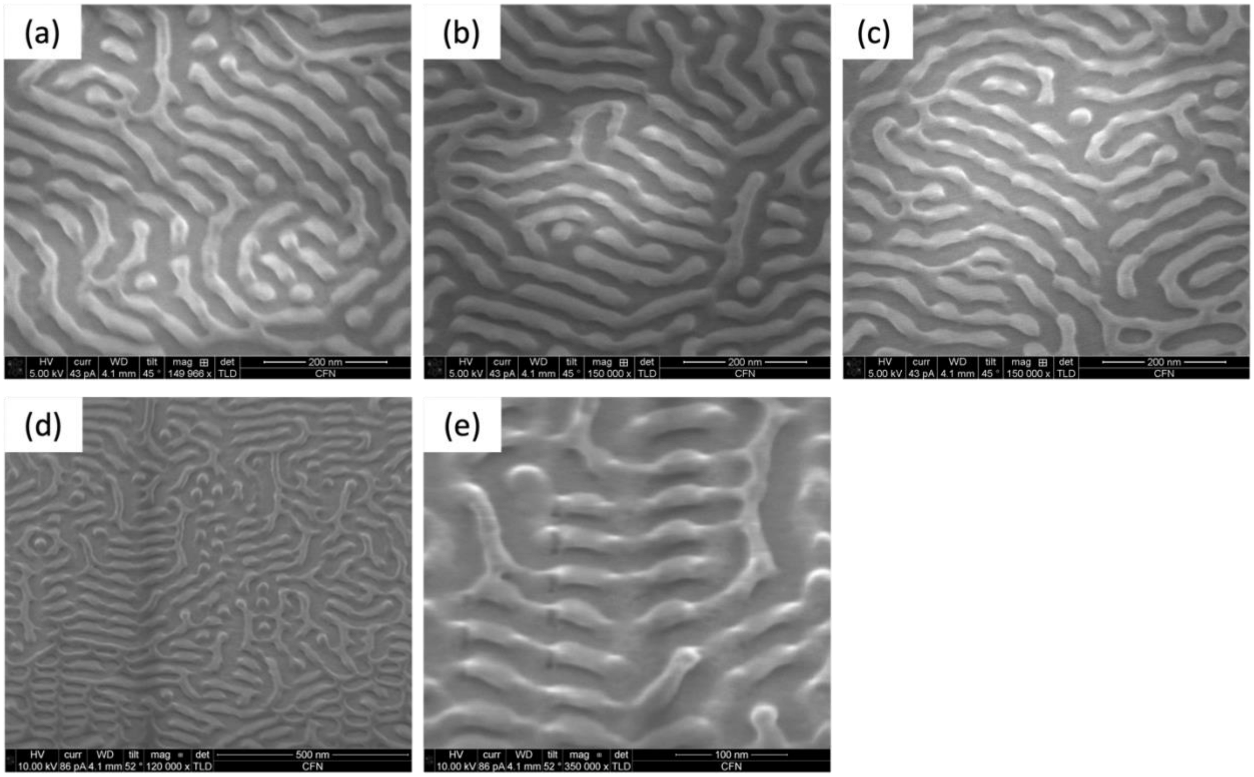

Fig. S9. Additional SEMs of morphologies observed in the skew region. Tilt view SEMs from the regions of the first (a-c; $\Lambda < \sim 70$ nm) and second skew populations (d,e; $\Lambda > \sim 70$ nm).

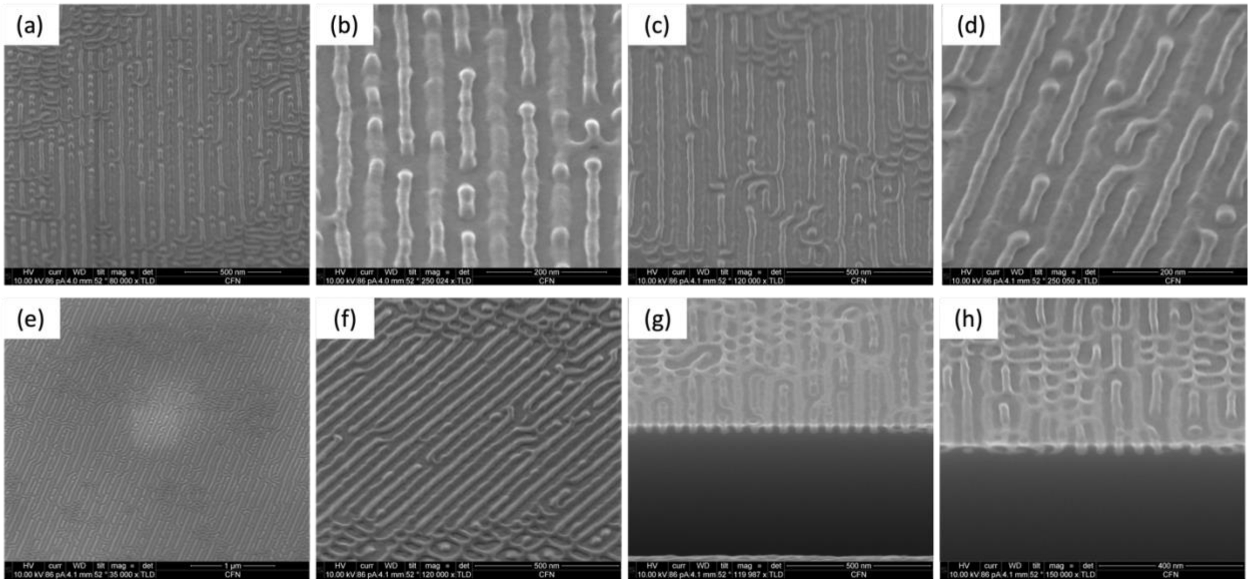

Fig. S10. Additional SEMs of the alternating subdomain morphology. Tilt view (a-f) and cross-section SEMs (g,h).

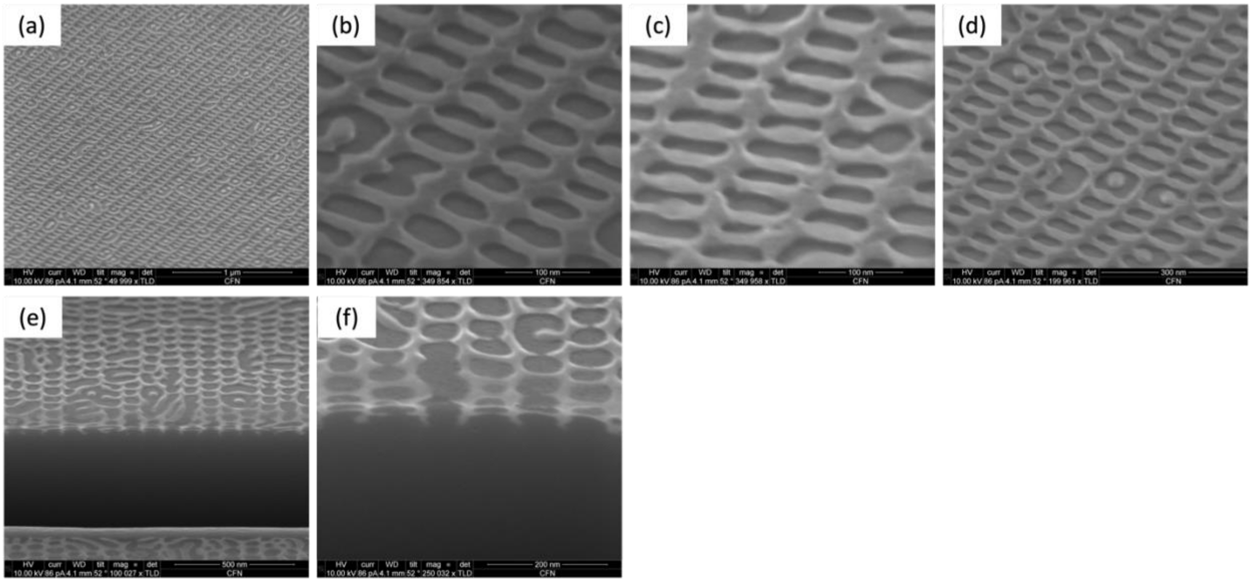

Fig. S11. Additional SEMs of the ladder morphology. Tilt view (a-d) and cross-section SEMs (e,f).

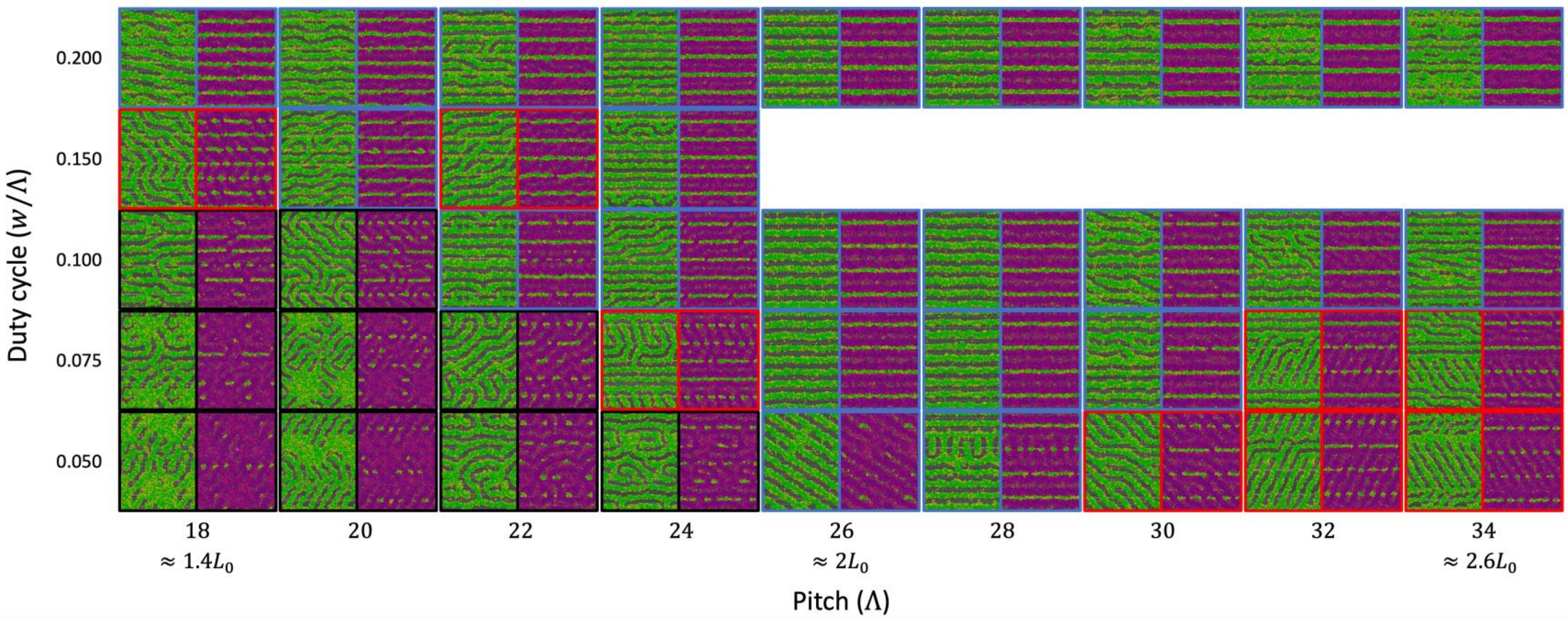
Duty cycle (w/Λ)
0.200
0.150
0.100
0.075
0.050
18
$\approx 1.4L_0$
20
22
24
26
$\approx 2L_0$
28
30
32
34
$\approx 2.6L_0$
Pitch (Λ)

Fig. S12. Coarse-grained molecular dynamics (MD) simulation results at film thickness $\boldsymbol{h \approx 0.9L_0}$ for a range of Λ from ~1.4 to 2.6$\boldsymbol{L_0}$ and duty cycles (*w*/Λ) from 0.05 to 0.2. For each simulation condition, the upper visualization is a top view with the majority block (pink/purple) removed. The lower visualization is a top view with both blocks at the template interface. Black borders denote a skew or island morphology, red borders denote a ladder-like or skew morphology, and blue borders denote an alternating morphology.

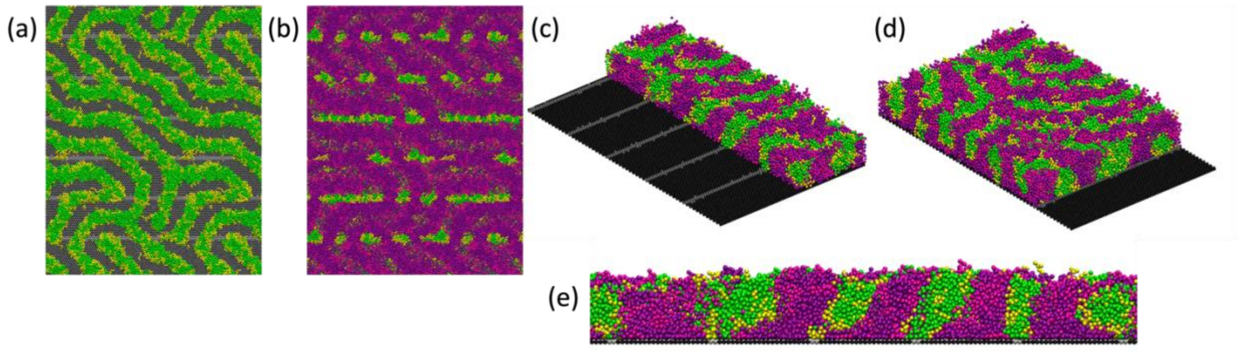

Fig. S13. MD results revealing a skew morphology at $h \approx 0.9L_0$, $\Lambda = 20\sigma \approx 1.54L_0$ and w/Λ = 0.150. Top view visualizations are shown with the majority block removed (a) or at the template interface (b). Three-dimensional tilt view visualizations are shown with cuts orthogonal to the template grating (c) and along a grating line (d). The minority domains contact the grating lines through vertical posts and connect with each other through overarching lines oriented at skew angles with respect to the grating direction. (e) A cross-sectional view of the cut shown in (c) shows that posts may slant in the vertical direction to maintain contact with the overarching lines.

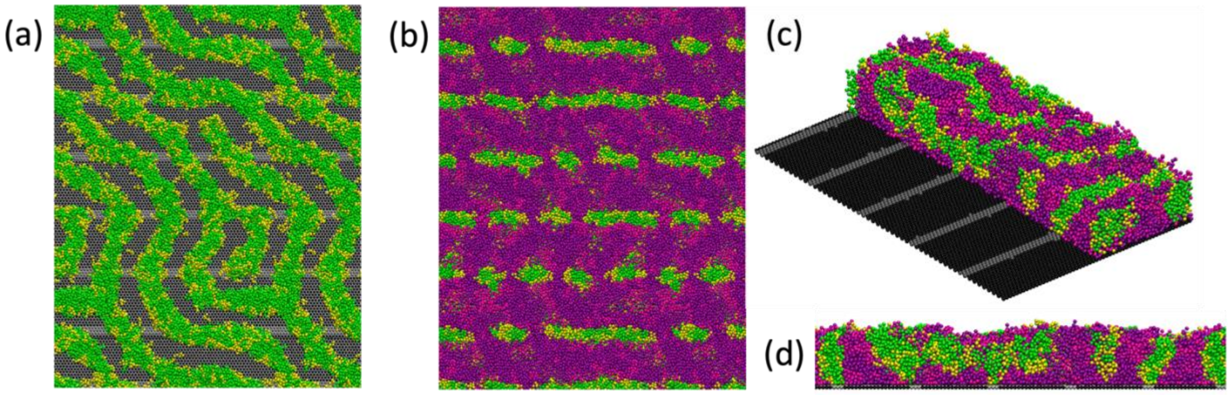

Fig. S14. MD results revealing a ladder-like morphology at $h \approx 0.9L_0$, $\Lambda = 18\sigma \approx 1.38L_0$ and *w*/Λ = 0.150. Top view visualizations are shown with the majority block removed (a) or at the template interface (b). Three-dimensional tilt view (c) and a cross-sectional (d) visualizations are shown with cuts orthogonal to the template grating. Minority domains contact the grating lines connect to each other via a suspended domain oriented nearly-orthogonally to the grating direction.

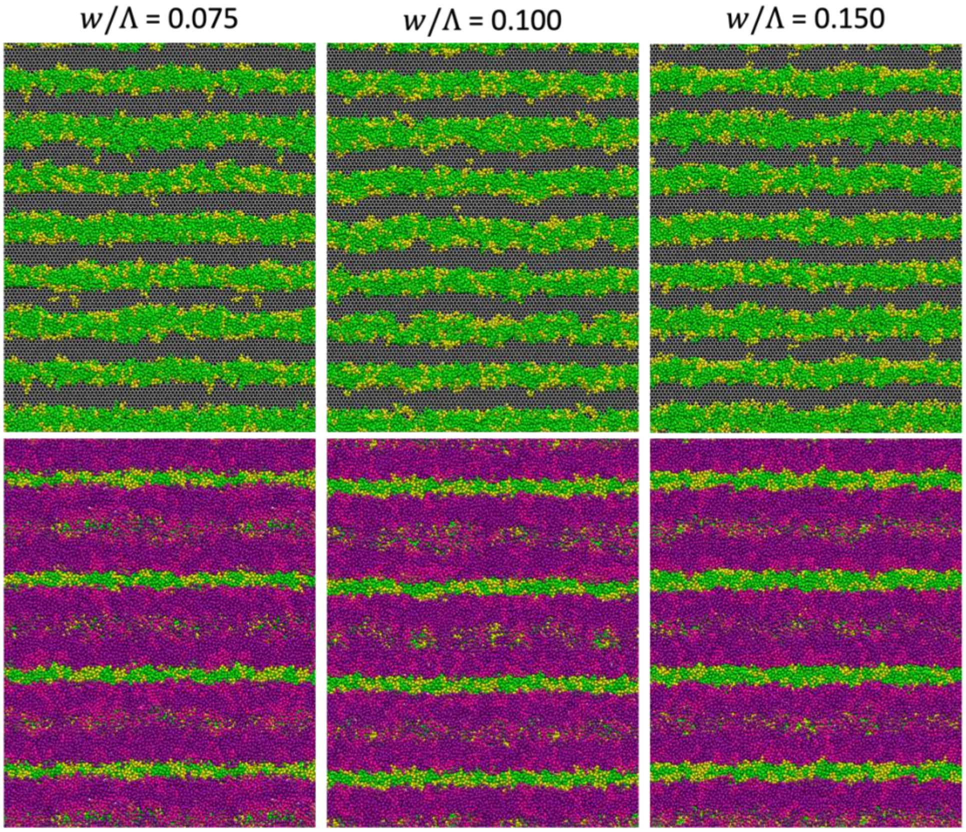

Fig. S15. MD results revealing an alternating morphology at $h = 0.75L_0$, $\Lambda = 26\sigma \approx 2L_0$ and various w/Λ. Top view visualizations are shown with the majority block removed (top row) or at the template interface (bottom row). The simulated film thickness matches the experimental film thickness, indicating the alternating morphology is prominent in the template region where it is experimentally observed ($\Lambda \approx 2L_0$ and large w).

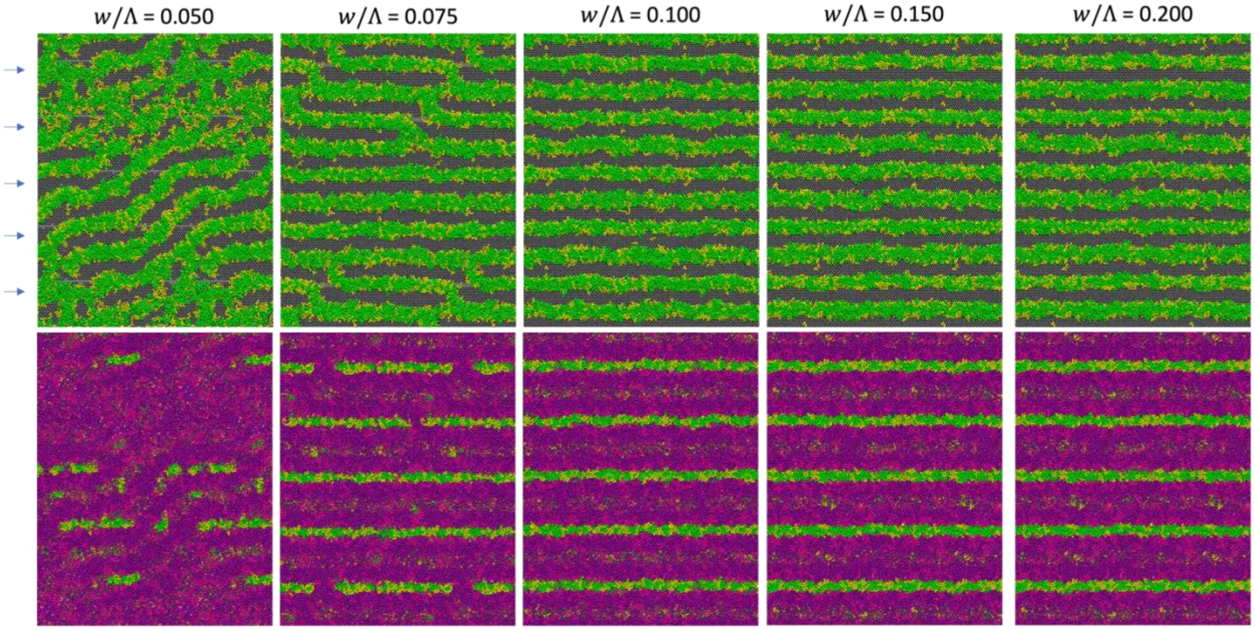

Fig. S16. MD results revealing an alternating morphology at $h \approx 0.7L_0$, $\Lambda = 24\sigma \approx 1.85L_0$ and various w/Λ. Top view visualizations are shown with the majority block removed (top row) or at the template interface (bottom row). Blue arrows at the left edge of the top row denote the position of the chemical grating lines. The relatively strong directing field imposed by the chemical pattern at this film thickness leads the alternating morphology to dominate down to w/Λ = 0.050, at which point a skew morphology emerges.

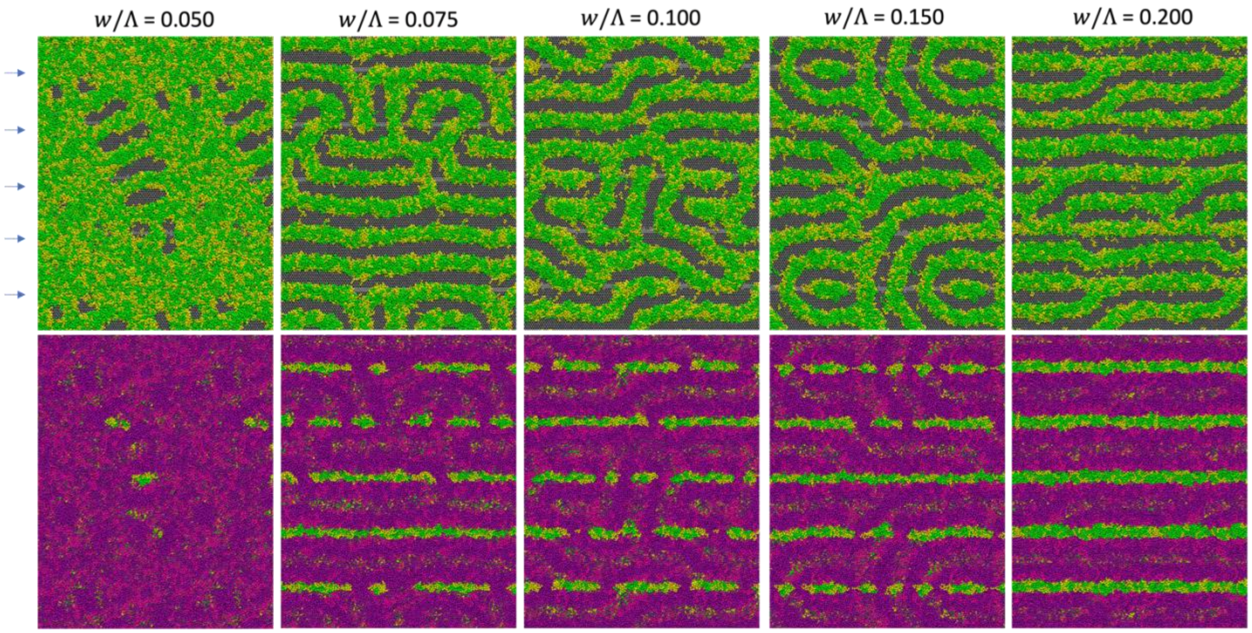

Fig. S17. MD results at $h \approx 1.25L_0$, $\Lambda = 24\sigma \approx 1.85L_0$ and various w/Λ. Top view visualizations are shown with the majority block removed (top row) or at the template interface (bottom row). Blue arrows at the left edge of the top row denote the position of the chemical grating lines. The weaker directing field imposed by the chemical pattern at this film thickness allows defects and alternative ladder-like morphologies to form, likely stabilized by localized BCP chain enrichment. Horizontal lamellae or island morphologies emerge at w/Λ = 0.050.

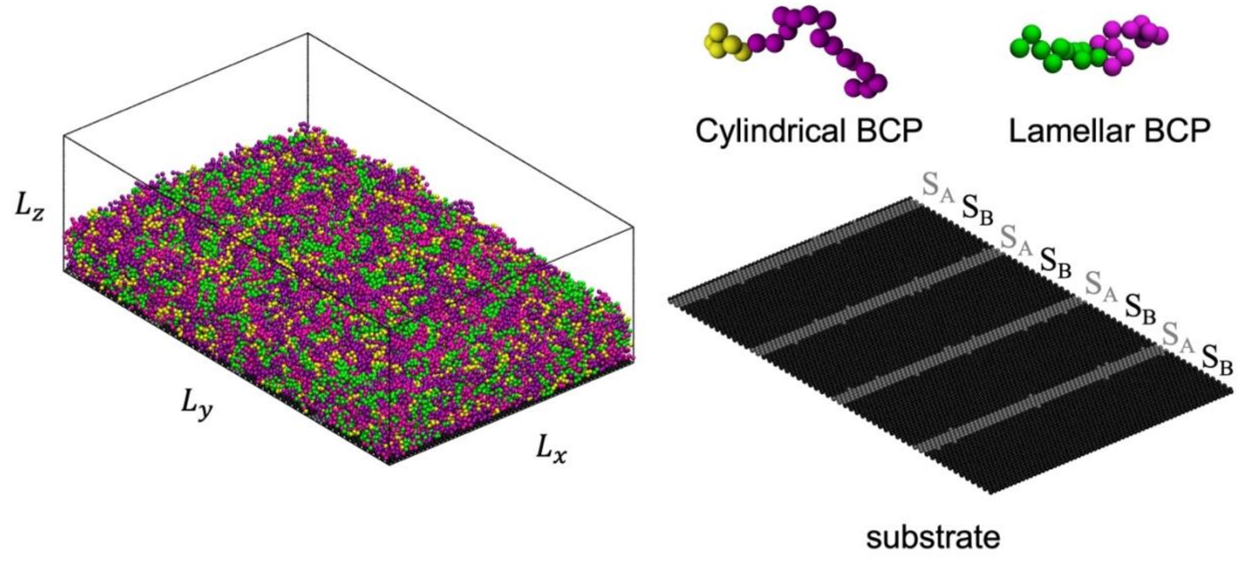

Fig. S18. MD simulation parameterization. (Left) The simulation box is defined with dimensions L_y (perpendicular to chemical grating), L_x (parallel to chemical grating), and L_z (vertical direction). Simulations are initiated using a film of randomly mixed cylindrical (asymmetric) and lamellar (symmetric) A-b-B BCP chains of thickness << L_z placed on top of a chemical pattern, which are subsequently allowed to equilibrate to the self-assembled morphology. (Right, top) The A-b-B chains each consist of 20 beads. Cylindrical "C" BCPs are comprised of 5 A beads (yellow) and 15 B beads (purple), while lamellar "L" BCPs are comprised of 10 A beads (green) and 10 B beads (magenta). (Right bottom) The underlying chemical pattern consists of a single layer of fixed beads with A bead stripes (S_{A}) of width w at a pitch $\boldsymbol{\Lambda}$ and a background (S_{B}) of fixed B beads.
